# Peroxidase Activity and Involvement in the Oxidative Stress Response of *Roseobacter denitrificans* Truncated Hemoglobin

**DOI:** 10.1371/journal.pone.0117768

**Published:** 2015-02-06

**Authors:** Yaya Wang, Xavier Barbeau, Astha Bilimoria, Patrick Lagüe, Manon Couture, Joseph Kuo-Hsiang Tang

**Affiliations:** 1 Department of Chemistry and Biochemistry, Clark University, Worcester, MA, United States of America; 2 Department of Biochemistry, Microbiology and Bioinformatics and PROTEO, Laval University, Quebec, Canada; Instituto de Tecnologia Quimica e Biologica, PORTUGAL

## Abstract

*Roseobacter denitrificans* is a member of the widespread marine *Roseobacter* genus. We report the first characterization of a truncated hemoglobin from *R. denitrificans* (*Rd*. trHb) that was purified in the heme-bound form from heterologous expression of the protein in *Escherichia coli. Rd*. trHb exhibits predominantly alpha-helical secondary structure and absorbs light at 412, 538 and 572 nm. The phylogenetic classification suggests that *Rd*. trHb falls into group II trHbs, whereas sequence alignments indicate that it shares certain important heme pocket residues with group I trHbs in addition to those of group II trHbs. The resonance Raman spectra indicate that the isolated *Rd*. trHb contains a ferric heme that is mostly 6-coordinate low-spin and that the heme of the ferrous form displays a mixture of 5- and 6-coordinate states. Two Fe-His stretching modes were detected, notably one at 248 cm^-1^, which has been reported in peroxidases and some flavohemoglobins that contain an Fe-His-Asp (or Glu) catalytic triad, but was never reported before in a trHb. We show that *Rd*. trHb exhibits a significant peroxidase activity with a (*k*_cat_/*K*_m_) value three orders of magnitude higher than that of bovine Hb and only one order lower than that of horseradish peroxidase. This enzymatic activity is pH-dependent with a p*K*_a_ value ~6.8. Homology modeling suggests that residues known to be important for interactions with heme-bound ligands in group II trHbs from *Mycobacterium tuberculosis* and *Bacillus subtilis* are pointing toward to heme in *Rd*. trHb. Genomic organization and gene expression profiles imply possible functions for detoxification of reactive oxygen and nitrogen species *in vivo*. Altogether, *Rd*. trHb exhibits some distinctive features and appears equipped to help the bacterium to cope with reactive oxygen/nitrogen species and/or to operate redox biochemistry.

## INTRODUCTION

Marine *Roseobacters* [1,2,3] are known to be abundant in the ocean ecosystem near the surface [4], and recent studies indicate that they are also responsible for producing reactive oxygen species in deep ocean, where light is limited or absent [5]. *Roseobacter denitrificans*, which may form a symbiotic relationship with marine eukaryotic communities as it was isolated from seaweeds in Tokyo Bay [6], is an aerobic anoxygenic (non-oxygen evolving) photosynthetic bacterium. It is one of the first *Roseobacters* being characterized [7] and the first *Roseobacter* species whose genome was sequenced [8]. The genomic information and phylogenetic analyses suggest that this obligate aerobic bacterium is closely related to some anaerobic photosynthetic α-*Proteobacteria*. As its name suggests, *R. denitrificans* contains genes required for denitrification and was reported to perform denitrification aerobically [9,10]. However, oxygen is known to control/repress denitrification, which is normally considered as an oxygen-limited or anoxic process employed by anaerobes for performing cellular respiration in the absence of oxygen. *R. denitrificans* can perform aerobic respiration with a complete electron transport chain, and electron transfer to O_2_ was shown to be energetically more favorable than to nitrate. Also, this bacterium cannot grow under strictly denitrifying conditions [10]. Thus the aerobic lifestyle of *Roseobacters* is not completely understood and the biological function of aerobic denitrification remains to be determined.

**Fig 1:**
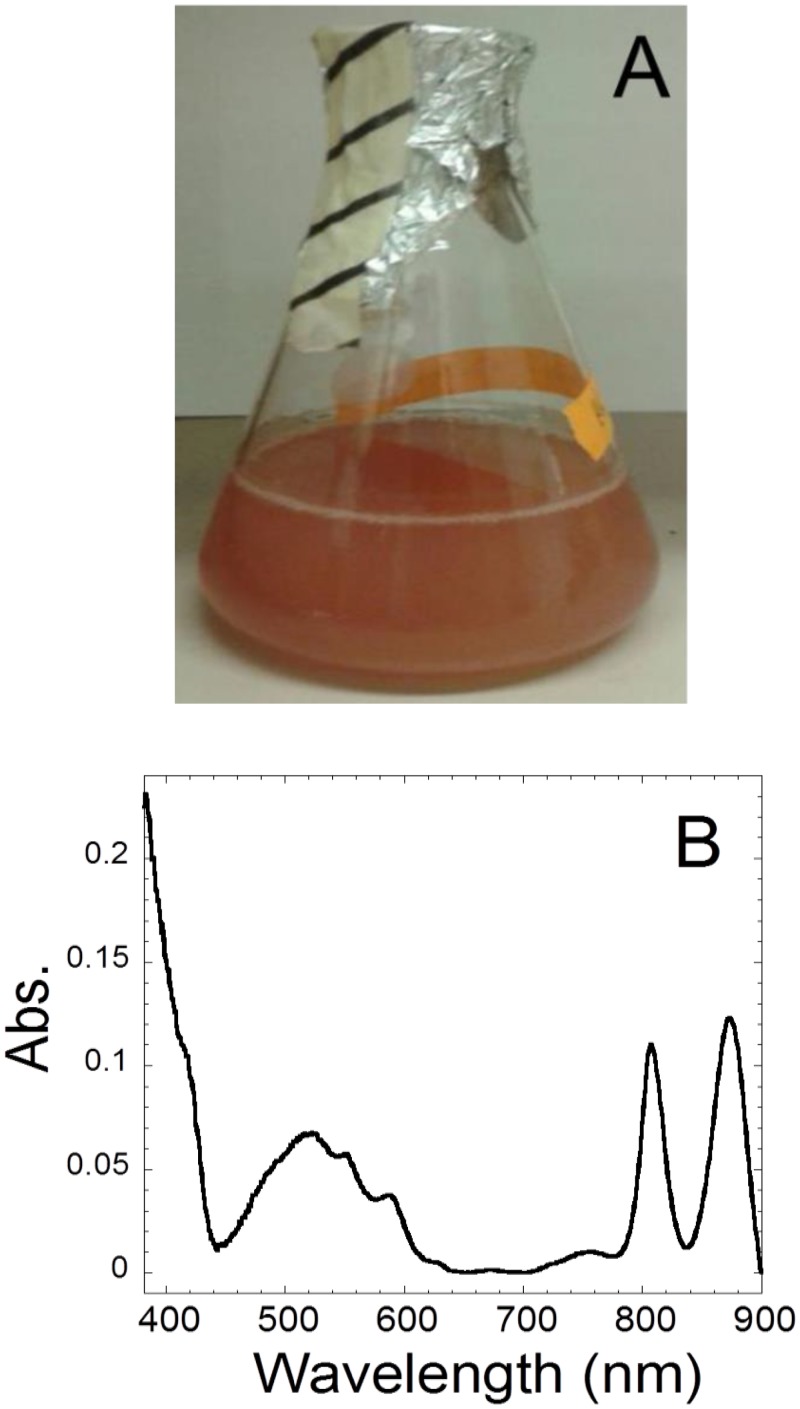
A cell culture of *R. denitrificans* (A) and its UV-visible absorption spectrum with baseline subtracted (B).

Cultures of *R. denitrificans* are very reddish as the name “*Roseo*”-*bacter* suggests (see Fig. 1). Optical absorption spectra of *R. denitrificans* cultures display wavelength maxima at 808 and 873 nm (light-harvesting antenna complexes), 450–600 nm (photosynthetic pigments) and ~400 nm. The latter indicates the presence of various hemoproteins, such as cytochromes and hemoglobins (Hbs). Cytochromes responsible for electron transport and redox reactions of *R. denitrificans* have been reported [11,12,13,14,15,16]. In addition, the genome of *R. denitrificans* [8] revealed the presence of a *glbO* gene encoding a putative a globin-like protein belonging to the truncated hemoglobins (trHbs) family of proteins, whereas globin-type proteins have not yet been reported in any member of the widespread *Roseobacter* clade.

TrHbs, which have primary amino acid sequences 20–40 residues shorter than full-length Hbs, have been identified in bacteria, unicellular eukaryotes and plants, but not in archaea and metazoan [17]. Three groups of trHbs, designated groups I, II and III, have been reported based on protein sequence analysis [17]. TrHbs have been identified in oxygenic phototrophs, which produce oxygen through water splitting during photosynthesis, including higher plants [18,19,20,21], cyanobacteria [22,23,24,25,26] and green algae [27,28], but have not yet been characterized in anoxygenic (non-oxygen evolving) phototrophs, such as *R. denitrificans*.

The atomic resolution structures of trHbs belonging to group I-III have been reported from many different organisms [29,30,31,32,33,34,35]. The oxygen carrier hemoglobin of metazoans has a very unique fold with a 3-over-3 alpha-helical sandwich motif, whereas the tertiary structure of trHbs is arranged as a 2-on-2 alpha-helical sandwich motif (S1 Fig.). Some trHbs display a unique hydrophobic cavity/tunnel system traversing the protein matrix from the molecular surface to the heme distal site [17]. Such a cavity/tunnel system may provide a path for diffusion of ligands/substrates/products to- and from the heme active site. The proposed functions of these proteins include nitric oxide detoxification, protection from reactive oxygen and nitrogen species (ROS/RNS), dioxygen scavenging and sulfide binding [34,36].

In this paper, we report the characterization of *R. denitrificans* trHb (*Rd*. trHb) obtained through sub-cloning of the *glbO* gene and heterologous expression of the recombinant protein in *E. coli*. This newly identified trHb was characterized by optical and resonance Raman spectroscopies and its peroxidase activity was investigated. We present sequence alignments of several trHbs from photosynthetic and non-photosynthetic microorganisms, as well as phylogenetic analyses of 46 trHbs from bacteria, unicellular eukaryotes and higher plants. Molecular modeling was employed to discuss possible structure/function relationships of *Rd*. trHb in comparison with other trHbs. Potential biological roles of *Rd*. trHb are proposed and the relation with the aerobic denitrification process by *R. denitrificans* is discussed.

## MATERIALS AND METHODS

### Materials

The DNA oligomers are from Integrated DNA Technology (IDT) and were used without further purification. All solvents and reagents were obtained from standard commercial sources and used as received. The chemicals, including hemoglobin (Hb) from bovine blood, horseradish peroxidase (HRP) and ABTS (2,2′-azino-bis(3-ethylbenzo-thiazoline-6-sulphonic acid)), were purchased from Sigma-Aldrich. H_2_O_2_ (30%, v/v) was from BDH Chemicals.

### Bacterial strains and culture conditions

*R. denitrificans* OCh 114 cells were grown in Difco^TM^ marina broth 2216 (Becton, Dickson and Company) [37] at 28°C with constant shaking at 180 rpm in 300 mL culture flasks with 100 mL of working volume. The cultures were grown to the stationary phase and harvested for extracting the genomic DNA using the protocol suggested in the QIAamp DNA Stool Mini Kit (Qiagen) with minor modifications.

### Cloning, expression and purification of recombinant *Rd*. trHb

The *R. denitrificans glbO* gene encoded a polypeptide of 152 amino acids, with a calculated molecular mass of 17.5 kDa. Polymerase chain reaction (PCR) was used to amplify the coding region of the *glbO* gene from the *R. denitrificans* genomic DNA with the method described previously [38]. DNA primers for gene amplification were: (forward primer, the *NcoI* site underlined) 5′-acctCCATGGcatgtgctttgcaaaagatgggcggc-3′ and (reverse primer, the *XhoI* site underlined) 5′-ACCTCTCGAGTCAGGCCCGCCGCAGCGCACCGTGA-TCC-3′. The *NcoI*/*XhoI*-digested fragment was inserted into *NcoI*/*XhoI*-cut pET-28a vector (Novagen/Merck) producing constructs for expression of *Rd*. trHb without N- or C-terminal His-tag. The ligation product was transformed to DH5α component cells (New England Biolabs) following the manufacturer’s protocol. Plasmid DNAs from a number of clones were isolated and screened for the presence of the insert by restriction digestion and DNA sequencing. The construct with the correct sequence was transferred to BL21(DE3) cells (New England Biolabs) for protein expression.

*E. coli* BL21(DE3) cells containing the constructed plasmid were grown at 37°C in 1-L flasks containing 500 mL Luria-Bertani (LB) medium and 50 μg/mL kanamycin until the absorption at 600 nm reached 0.6–0.8, at which time, the temperature was reduced to 16°C and the cells were incubated overnight to maximize protein expression. IPTG was not added to induce protein synthesis because it was found to inhibit cell growth. The cells were harvested by centrifugation at 5000 x g for 20 min at 4°C, resuspended in a lysis buffer containing 1 mM EDTA, 2 mM NaCl, 1 mM β-mercaptoethanol and 0.1% Triton X-100 in 20 mM Tris-HCl at pH 7.8. The cells were sonicated for 30 min on ice and then centrifuged at 5000 x *g* for 20 min. The soluble proteins were collected for further purification on a 0.7 x 15 cm FLEX column packed with Q-Sepharose Fast-Flow (GE Healthcare Life Sci.) (130 mL) and equilibrated with buffer A (20 mM Tris-HCl pH 8.0) containing 50 μM EDTA at 4°C. After washing, the proteins were eluted with buffer A containing 150 mM NaCl. The proteins were concentrated by ultrafiltration (10 kDa MWCO) and then dialyzed against buffer A containing 50 mM NaCl overnight at 4°C in 8 kDa MWCO membranes (Spectra/Por). The protein mixture was then loaded on a Hi-load 16/60 Superdex 75 gel-filtration column (GE Healthcare Life Sci.) equilibrated with 50 mM NaCl in buffer A at 4°C. The purified *Rd*. trHb was estimated to be a monomer based on the elution volume from the gel-filtration column. A total of 0.2–0.25 mg protein was isolated from 1-liter growth medium. The protein sequence of the heterologously expressed *Rd*. trHb was confirmed by peptide-mapping via mass spectrometry [39]. The protein concentration was determined by the Bradford assay and by the A_280_ using an extinction coefficient 15,470 M^-1^cm^-1^ calculated from the deduced amino acid sequence of *Rd*. trHb. To obtain the ferrous deoxygenated state of *Rd*. trHb, the purified ferric protein was made anaerobic by flushing with N_2_ and then reduced with 1.0% sodium dithionite.

### Optical spectral measurements

The UV-visible absorption spectra were recorded using a Shimadzu UV-1800 spectrophotometer. The CD spectra for *Rd*. trHb in a 1.5 mm path length quartz cuvette were recorded between 195 and 265 nm at 25°C using a Jasco J-810 CD spectrometer. All spectra were collected at 25°C.

### Peroxidase activity assays

The peroxidase activity was assayed using H_2_O_2_ as the oxidizing substrate and ABTS as the organic substrate. The reaction was first verified with HRP and monitored by the increase of absorbance at 414 nm (A_414_). The *Rd*. trHb-dependent H_2_O_2_-reduction was performed with 0.5 mM H_2_O_2_, 0.5 mM ABTS and various concentrations of *Rd*. trHb in 20 mM Tris-HCl buffer at pH 8.0. The concentration of the H_2_O_2_ stock solution was determined from the absorbance at 240 nm using an extinction coefficient = 43.5 M^-1^cm^-1^. The initial rates were determined in the initial linear phase of the progression curves. The concentration of oxidized ABTS was calculated from the increase at A_414_ using a molar extinction coefficient of 36,800 M^-1^cm^-1^ [40]. The steady-state kinetic parameters of HRP, *Rd*. trHb and bovine Hb were investigated using 13.5 nM HRP, 50 nM *Rd*. trHb and 77.5 nM bovine Hb and with various concentrations of H_2_O_2_ and 1 mM ABTS. The initial rates versus H_2_O_2_ concentration curves (6 to 7 H_2_O_2_/ABTS concentrations) were fitted to the Michaelis-Menten equation using the KaleidaGraph software to obtain apparent *k*_cat_ (*k*_cat,app_) and apparent *K*_m_ (*K*_m,app_) values for the different enzymes and for reactions followed at different pHs. The pH dependence of the enzymatic activity was measured using the following buffers: MES (pH 6.0), phosphate (pH 6.1–6.8), MOPS (pH 7.0), Tris-HCl (pH 8.0), glycine (pH 9.0) and NaHCO_3_ (pH 10.5). The pH dependences of *k*_cat,app_ and *K*_m,app_ were fitted to eq. 1:

$$k_{\text{cat,app}}or_{}K_{\text{m,app}}=\frac{{\left(k_{\text{cat,app}}\right)}_{\text{max}}\text{ or}\;{\left(K_{\text{m,app}}\right)}_{\text{max}}}{{\text{10}}^{\text{(pH-p}K\text{a)}}\text{ + 1}}$$ (1)

The pH dependence of *k*_cat,app_/*K*_m,app_ was fitted to eq. 2:

$$\frac{k_{\text{cat,app}}}{K_{\text{m,app}}}=\frac{{\left(\frac{{}_{\text{cat,app}}}{K_{\text{m,app}}}\right)}_{\text{min}}+{\left(\frac{k_{\text{cat,app}}}{K_{\text{m,app}}}\right)}_{\text{max}}({\text{10}}^{\text{(p}K\text{a-pH)}})}{{\text{10}}^{\text{(p}K\text{a-pH)}}\text{ + 1}}$$ (2)

### Resonance Raman spectroscopy

Samples for Raman spectroscopy were used at ~50 μM concentration in 20 mM Tris-HCl at pH 8 buffer containing 50 mM NaCl. To record the resonance Raman spectrum of the ferric state, the protein was used as purified. This sample did not contain protein in the oxygenated state as judged from the absence of any molecular oxygen isotope sensitive line (determined with ^18^O_2_, 99%, Icon Isotopes). The reduced and exogenous ligand-free protein was obtained by flushing the ferric protein with Ar in a tightly sealed custom-made Raman cuvette and by adding a small amount of a freshly prepared sodium dithionite solution. To obtain the Fe(II)CO complex, the ferric protein was first flushed with Ar, then ^13^C^18^O (99% ^13^C; 95% ^18^O, Icon Isotopes) was added with a gas-tight syringe and the heme was reduced with dithionite. To acquire the resonance Raman spectrum with ^12^C^16^O, the Fe(II)^13^C^18^O sample was flushed with ^12^C^16^O a few minutes to exchange the CO molecules. The equipment used to acquire the resonance Raman spectra has been previously described [41]. The ferric, reduced and Fe(II)CO states were investigated with the 413.1 nm line from a krypton ion laser. The reduced state was also investigated with the 441.6 nm line from a He/Cd laser. The laser power on the samples was kept at less than 2 mW. The spectrometer was calibrated with the lines of indene in the low- and high-frequency regions. Cosmic ray lines were removed from the spectra by a routine of the Winspec software used for data acquisition (Roper Scientific, Princeton, NJ). Several spectra were acquired over a period of 30 minutes, averaged and analyzed using the Grams/AI software (ThermoGalactic).

### Molecular modeling

The primary sequence of *Rd*. trHb (152 residues) was obtained from the UniProt Consortium (UniProt Consortium, 2012) (UniProt Q160B8). Three structures were identified as templates for homology modeling from a standard protein Blast (blastp) query using the Protein Data Bank (PDB) database on the NCBI/Blast web server (http://blast.ncbi.nlm.nih.gov/Blast.cgi). The three truncated hemoglobin structures identified are from *Bacillus subtilis* [42], *Arabidopsis thaliana* [43] and *Agrobacterium tumefaciens* [44], with UniProt identification numbers O31607, Q67XG0 and Q7CX73, respectively. A multiple sequence alignment of these sequences using the default parameters of Muscle v3.8.31 [45,46] showed percentage of similarity of 50.0, 48.0 and 38.5% and percentage of identity of 32.2, 27.7 and 28.3%, between the trHbs of *R. denitrificans* and *B. subtilis*, *A. thaliana* and *A. tumefaciens*, respectively. The crystal structure of *B. subtilis* trHb (PDB ID 1UX8) was selected as the template because it presented the highest percentage identity with *Rd*. trHb. However, the sequence in *Rd*. trHb had an insertion of 4 residues between the B and C helices. To better model this region, the BC-loop section from *B. subtilis* trHb (PDB ID 1UX8) was replaced by corresponding backbone sections from *A. thaliana* trHb (Glb3) (PDB ID 4C0N) and *A. tumefaciens* trHb (PDB ID 2XYK), leading to 33.6% of identity and only 1 gap in the primary sequence alignment of the C-helix region. The model was built with the resulting scaffold using the Muscle multiple sequence alignment in MOE (Molecular Operating Environment) [47], using the CHARMM27 force field and the born implicit solvation model. Twenty-five backbone variations, each with twenty-five side chain positions, were explored. The best model was selected based on the GB/SI score in MOE and was validated with the MolProbity web server [48]. The heme cofactor from the *B. subtilis* structure was then added to the model, protonated and minimized with MOE.

### Cultures prepared for gene expression assays

For investigating the gene expression profiles, *R. denitrificans* were grown in minimal medium supplied with 20 mM glucose as the sole carbon source and subjected to light/dark cycles (12 h:12 h) or with 20 mM acetate as the sole carbon source in darkness [37]. Reagents in 1-L minimal medium (pH 7.5) were: 3.2% (w/v) Instant Ocean sea salt, 0.1 g MgSO_4_, 0.1 mM FeCl_3_•6H_2_O, 0.05 g Na_2_HPO_4_, 0.5 g KNO_3_, 0.1 g CaCl_2_•2H_2_O, 0.12 g Tris, 1.0 mg thiamine-HCl, 1.0 mg nicotinic acid, 1.0 mg Na-pantothenate, 0.1 mg biotin, 0.5 mg vitamin B_12_ and 1.0 mL trace elements solution (3.0 g FeCl_3_•6H_2_O, 0.1 g MnSO_4_•H_2_O, 50 mg H_3_BO_3_, 50 mg CuSO_4_•5H_2_O, 50 mg NaMoO_4_•2H_2_O, 0.1 g ZnCl_2_, 0.5 g Na_2_EDTA, 0.2 g CaCl_2_• 2H_2_O, 0.1 g CoCl_2_•6H_2_O, 50 mg NiCl_2_•6H_2_O in 1-liter H_2_O) [7,37]. Phototrophic cultures were illuminated with low-intensity light (60 μmole/m^2^/s). Cultures in the mid-log growth phase were chosen for gene expression analyses.

### RNA extraction and quantitative real-time polymerase chain reaction (QRT-PCR)

RNA was isolated from cell pellets using the TRIzol reagent (Invitrogen) according to the manufacturer’s protocol, and DNase was used to remove DNA. The absence of contamination of the RNA samples by genomic DNA was verified by PCR and agarose gel electrophoresis. QRT-PCR was carried out to profile the gene expression levels under different growth conditions. cDNA was synthesized from 1 μg of RNA and 100 μM random 9-mer DNA using the Superscript III reverse transcriptase (Invitrogen). QRT-PCRs were performed with the Mx-3000P qPCR systems (Agilent Technologies, Inc.). The primers for QRT-PCRs (shown in Table 1) were designed with the program Primer3 (http://www.ncbi.nlm.nih.gov/tools/primer-blast/) and analyzed with Oligo-Analyzer 3.0 (Integrated DNA Technologies). The Power SYBR green master mix (Qiagen) was used to amplify DNA with the following cycles: an initial denaturation step (15 min at 95°C), followed by 40 amplification cycles (15 s at 95°C, 30 s at 60°C and 45 s at 72°C) and then 1 dissociation cycle (15 s at 95°C, 1 min at 60°C and 15 s at 95°C). The threshold cycle (C_T_) was calculated as the cycle number at which ΔRn crossed the baseline. 16S rRNA was used as the internal control gene transcript. The following parameters were calculated: ΔC_T_ that corresponds to C_T_ (target gene)—C_T_ (16S rRNA), ΔΔC_T_ that corresponds to absolute value of ΔC_T_ in two different culture conditions and the relative expression level that corresponds to 2^(absolute value of ΔΔCT)^ [49]. Three biological replicates, with three technical replicates for each biological sample, were analyzed and the mean value was reported. The amplified DNA fragments were verified by agarose gel electrophoresis.

**Table 1:** Primer sequences used in this report.

Gene locus	Primer sequences (5′ to 3′)	Gene product
16S rRNA	Forward: tgttcggaattactgggcg	16S rRNA
gene	Reverse: tcgggatttcacccctaactt	
	Glycogenesis and gluconeogenesis	
RD1_2870	Forward: tgcagccatttgtgaaacat	Phosphoglucomutase
	Reverse: atgcgttttgtgatgtcgaa	
RD1_2720	Forward: cagtgacgttacggatgtgg	Glucose-6-phosphate
(pgi)	Reverse: aatggtcgtgaaggtcttgg	isomerase
	Carbohydrate catabolism	
RD1_2879	Forward: CGCACGGTGCTTTTTTCG	6-phosphogluconate dehydrase
(edd)	Reverse: GTTCCTGCCAGCGGGTC	
RD1_2878	Forward: CCAGAAGTGGTAATTCCAGCG	2-dehydro-3-deoxy-phospho-
(eda)	Reverse: TTCACCCGGCGCGAC	gluconate aldolase
	Carbon assimilation	
RD1_3376	Forward: CCTTGGGCTTGCGGATC	Pyruvate carboxylase
(Pyc)	Reverse: CATCTGGTTCACCTCGGCA	
RD1_0421	Forward: ACCCCCGGAAAGTTCGAG	Malic enzyme
(Tme)	Reverse: AAGACTGAGGTCCCGCTGC	
	The tricarboxylic acid (TCA) cycle	
RD1_1609	Forward: TTCGGGCAAGGTCTATTACG	2-oxoglutarate dehydrogenase
(sucA)	Reverse: CTTGGGTGTTTGGCTTTGAT	
RD1_2204	Forward: TCTTCTGGCTCGACGAAGAT	Isocitrate dehydrogenase
(Icd)	Reverse: GATGTGCCCAACTCAAGGAT	
	Nitrogen metabolism	
RD1_1561	Forward: tgaccgtgagatcatcgaaa	Nitric oxide reductase
(norB)	Reverse: aaccatgacaaaggcaaagg	
RD1_1562	Forward: tttcggtccattcacacaga	Nitric oxide reductase
(norC)	Reverse: tgtcatcacattgcccagtt	
	Catalase and peroxidases	
RD1_2195	Forward: GCCTGACTTCTTCGTCAACC	Bi-functional catalase/peroxidase
(katG)	Reverse: GAATTTTTCGGCGTTGTCAT	
RD1_0599	Forward: TCTGCGAAATGAACTTTGGA	Glutathione peroxidase
(gpo)	Reverse: ATTGGTTTTGTCTGCGAACC	
	Function(s) yet unidentified	
RD1_4240	Forward: gacatcatggaaaccgatcc	Truncated hemoglobin
(glbO)	Reverse: atctcacgcaggttcatgtg	

### Phylogenetic tree

A phylogenetic tree was constructed based on the primary sequences of 46 trHbs with the Neighbor-Joining method [50] using the phylogenetic software MEGA6 [51]. The percentage of replicate trees for which the associated taxa clustered together in the bootstrap test (1000 replicates) are shown next to the branches. The tree is drawn to scale, with branch lengths in the same units as those of the evolutionary distances used to infer the phylogenetic tree. All positions containing gaps and missing data were eliminated. The final data set contained 88 positions.

## RESULTS AND DISCUSSION

### Optical spectra of the recombinant *Rd*. trHb

**Fig 2:**
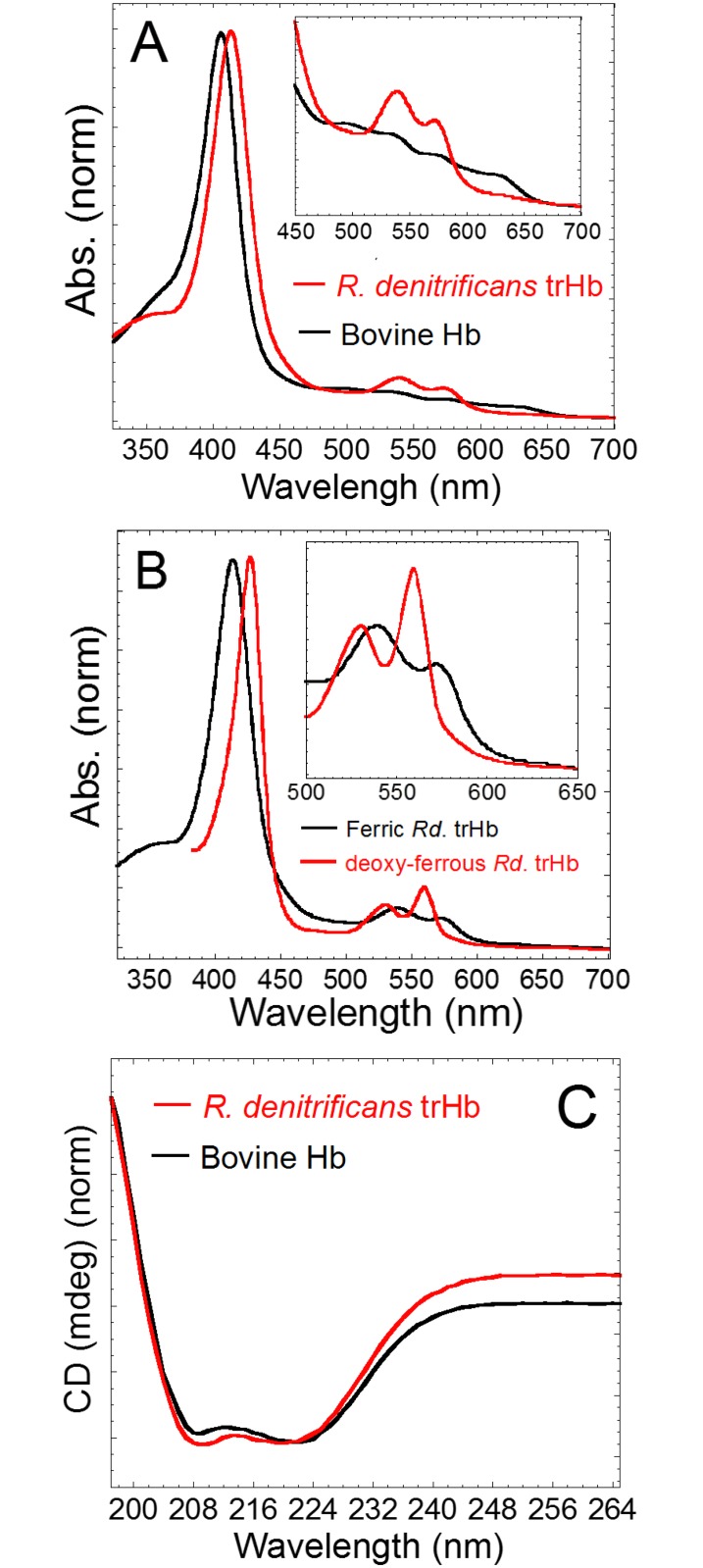
The UV-visible absorption and CD spectra of bovine Hb and of the recombinant *R. denitrificans* trHb. The absorption spectra of ferric *R. denitrificans* trHb and bovine Hb (A). The absorption spectrum of *Rd*. trHb in the ferric and deoxygenated ferrous states (B). The CD spectra of *R. denitrificans* trHb and bovine Hb (C). The inset in panels (A) and (B) shows the Q bands region. Samples were prepared in 50 mM NaCl in 20 mM Tris-HCl at pH 8.0 and the spectra were recorded at 25°C.

The *glbO* gene encoding trHb from *R. denitrificans* (*Rd*. trHb) was sub-cloned and the recombinant protein was heterologously expressed in *E. coli* and purified using the protocol reported for a group II trHb from *Mycobacterium tuberculosis* (*Mt*. trHbO) [52] with minor modifications. The recombinant *Rd*. trHb appears to be red (not shown), implying that the purified protein contained heme. Fig. 2 shows the UV-visible absorption and circular dichroism (CD) spectra of recombinant *Rd*. trHb and of bovine Hb (i.e. a “full-length” Hb). The UV-visible absorption spectrum of *Rd*. trHb (Fig. 2A) is typical for a heme-bound protein with a characteristic Soret band (412 nm). The heme appears to be six-coordinate and low-spin heme with Q bands at 538 and 572 nm. We have also isolated a trHb from *R. denitrificans* cultures. This native protein displayed the same optical absorption spectrum (data not shown). In contrast, ferric bovine Hb exhibits a mixture of high-spin and low-spin states with a Soret band at 406 nm and Q bands at 497, 537, 573 and 630 nm. The absorption spectrum of *Rd*. trHb in the deoxygenated ferrous state shows the spectral features of a hexa-coordinate heme, with two Q bands at 532 and 560 nm [53,54] (Fig. 2B). The CD spectra (Fig. 2C) show that *Rd*. trHb, like bovine Hb, largely contains alpha-helical secondary structure elements, in agreement with the 2-on-2 alpha-helical sandwich structure reported for trHbs [29,30,33].

### Sequence alignment and phylogenetic analysis

**Fig 3:**
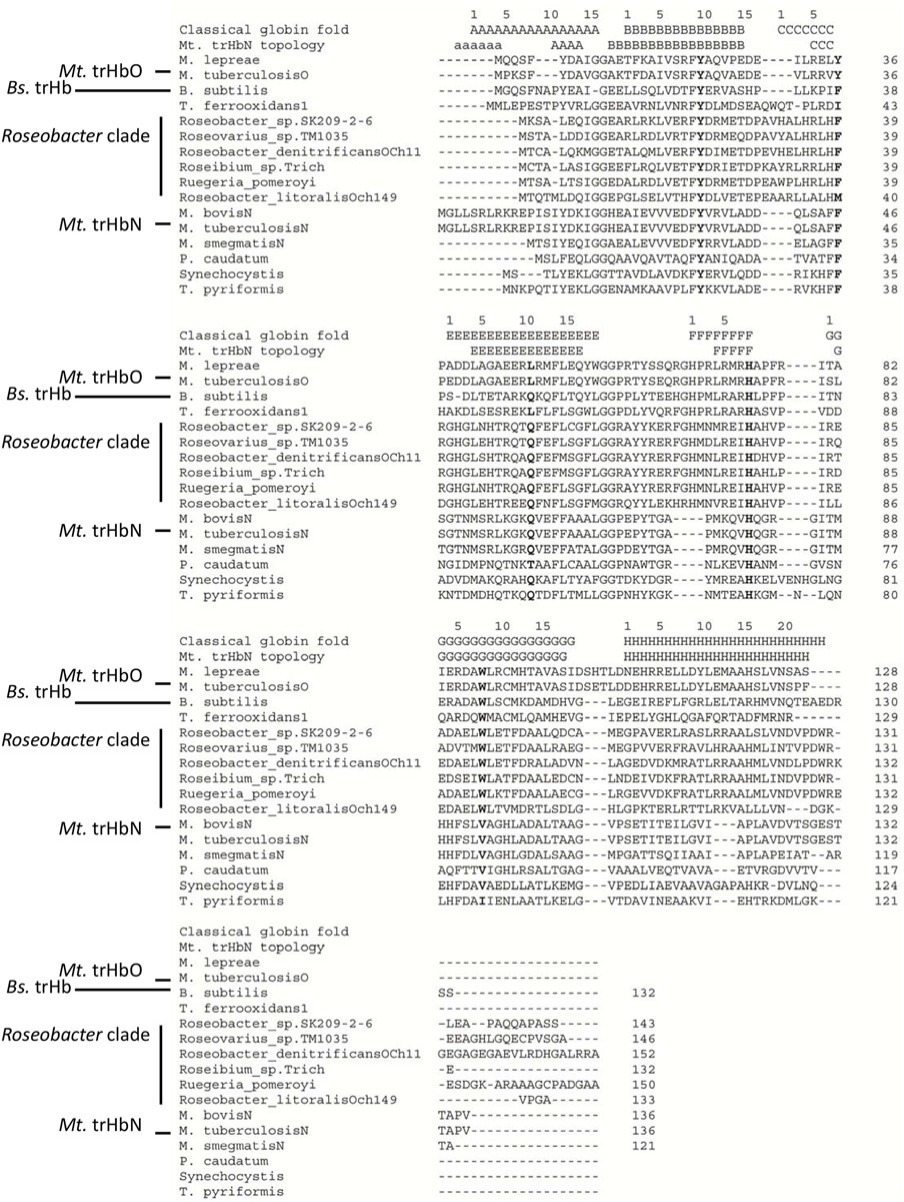
Multiple sequence alignment of selected trHbs. Topological sites referring to the classical globin fold are indicated on top of the multiple sequence alignment (A, B, C, E, F, G and H) [30]. The secondary structure elements based on the structure of trHbN from *Mycobacterium tuberculosis* are also indicated above the sequences (the helix labelled in lower case refers to the pre helix-a region of the N-terminus of *Mt*. trHbN). Amino acids at key sites are indicated in bold (B10, CD1, E11, F8 and G8). Sequence accession numbers: *M. leprae* (trHbO), WP_010908224; *M. tuberculosis* (trHbO), WP_003899335; *T. ferrooxidans*, WP_012607088; *R. denitrificans* OCh 114, WP_011570285; *R. trich* SKD4, WP_009760112; *R. pomeroyi* DSS-3, YP_166804; *Roseovarius* sp. TM1035, WP_008280790; *R. litoralis* OCh 149, WP_013960546; *M. bovis* (trHbN), P0A593; *M. tuberculosis* (trHbN), P9WN25; *M. smegmatis* (trHbN), WP_011730775; *P. caudatum*, P15160; *T. pyriformis*, P17724; *Synechocystis* sp. PCC 6803, WP_010872616; *B. subtilis*, O31607; *Roseobacter* sp. SK209–2–6, EBA15453.

The multiple protein sequence alignment (Fig. 3) shows that *Rd*. trHb has the conversed His at F8 (His being the proximal ligand to the heme in globins) and Trp at G8 (important for O_2_ binding and stabilization in *Mt*. trHbO (*vide infra*) [55], lacks Tyr at CD1 (with Phe instead) but has Gln at E11 and Tyr at B10 that are the important amino acids of group I *Mt*. trHb (*Mt*. trHbN) [30]. The percentages of identity and similarity were 16% and 27% between *Rd*. trHb and *Mt*. trHbN, respectively. Although the phylogenic analyses suggest that *Rd*. trHb falls in group II trHbs, which include *Mt*. trHbO, it has an interesting mix of potential heme pocket distal amino acids distinct from those of *Mt*. trHbO but similar to those of the group II trHb of *Bacillus subtilis* (*Bs*. trHbO) [56].

**Fig 4:**
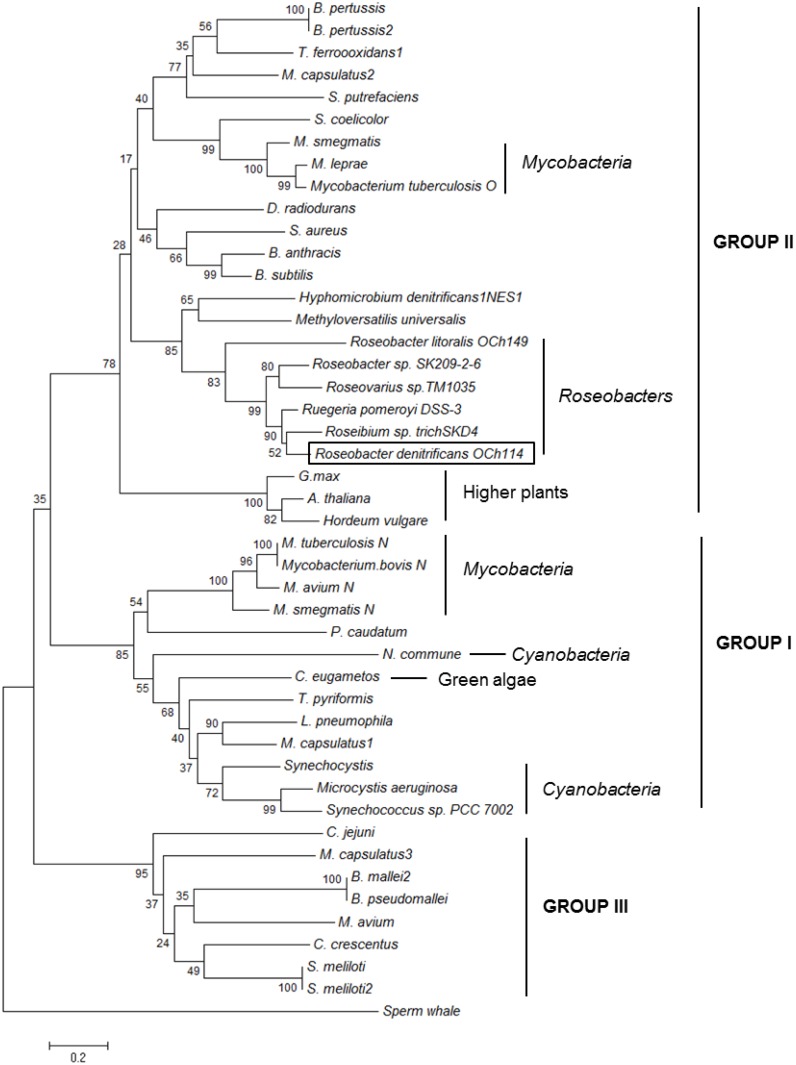
The phylogenetic tree of trHbs. The evolutionary history was inferred using the Neighbor-Joining method. Sperm whale myoglobin was used as the out-group. The analysis involved 46 amino acid sequences. All positions containing gaps and missing data were eliminated. Evolutionary analyses were conducted using the software MEGA6 [51]. Three groups (groups I, II and III) of trHbs are identified.

The phylogenetic tree built from 46 well characterized and putative trHbs from different organisms for illustrating evolutionary relationships among trHbs is shown in Fig. 4. TrHbs from cyanobacteria and green algae are identified as group I trHbs. TrHbs from *R. denitrificans* and several members of the *Roseobacters* genus are identified as group II trHbs and thus fall into the same group of *Mt*. trHbO [57] and *Bs*. trHbO [42] along with trHbs of higher plants.

### Molecular modeling

**Fig 5:**
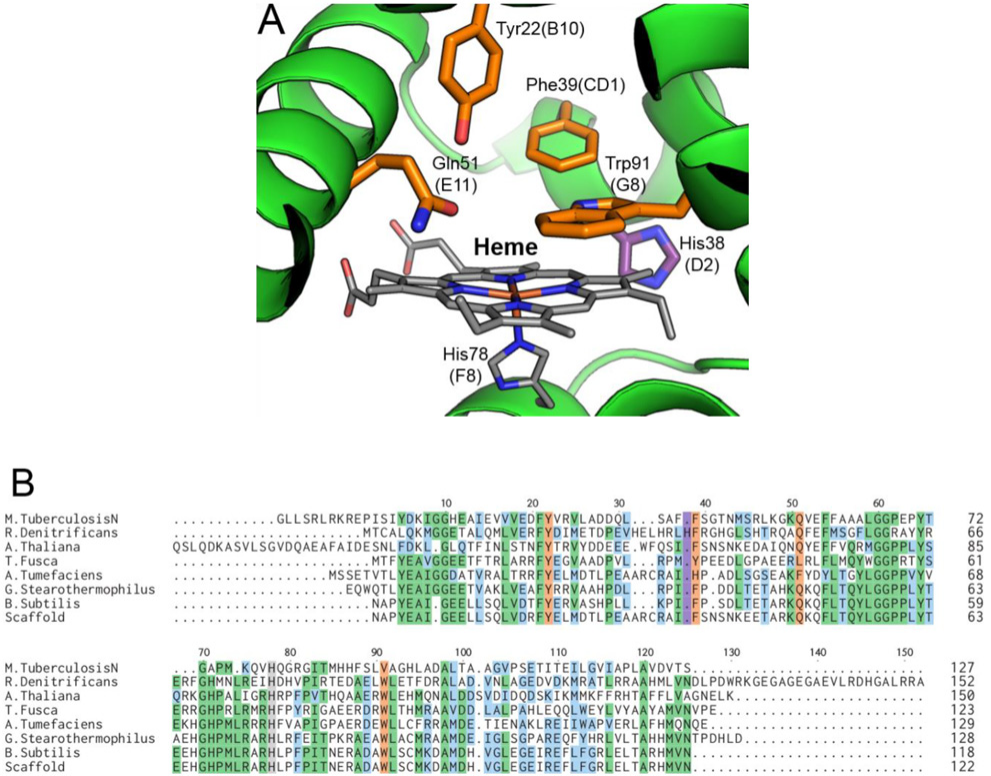
Molecular modeling of *Rd*. trHb. Structural representation of the active site from the homology model of *Rd*. trHb (green cartoon). His78 at the position F8 (the proximal His), which coordinates the heme, is highlighted in grey sticks. His38 at position D2, unique to *Rd*. trHb, is highlighted in purple. Important residues of the active site of *Rd*. trHb are highlighted in orange. Oxygen atoms are in red, nitrogen in blue and the Fe atom is in orange (A). The primary sequence alignment of trHbs from *M. tuberculosis*, *R. denitrificans*, *A. thaliana*, *A. fusca*, *A. tumefaciens*, *G. stearothemopilus* and *B. subtilis* is shown. Conserved residues are colored in green, similar residues in blue, diverging residues in white and the proximal His in grey. His at the position D2, unique to *Rd*. trHb, is highlighted in purple and important catalytic residues of the active site are in orange. Numbers showed on top are those of the *Rd*. trHb primary sequence and numbering for each sequence is showed on the right. The constructed template used for homology modeling is denoted as Scaffold (B).

The homology model of *Rd*. trHb was built using the *Bs*. trHbO crystal structure, except for a 4-residue insertion in the D-helix that was modeled based on the *A. thaliana* trHb and *A. tumefaciens* trHb crystal structures (see Materials and Methods for details). The distal site of the homology model of *Rd*. trHb is presented in Fig. 5A and the sequence alignment used for generating the model is shown in Fig. 5B. Fig. 5A shows that the highly conserved residues Tyr22(B10), His38(CD region), Phe39(CD1), Gln51(E11) and Trp91(G8) are found in the distal site pointing toward to the heme, and His78(F8) is the proximal His coordinating to the Fe. In *Mt*. trHbN, *Mt*. trHbO and *Bs*. trHbO, the residues involved in polar interactions with heme-bound ligands were described as a ligand-inclusive network of hydrogen bond interactions [42,56,58]. The amino acids involved in ligand stabilization are Tyr(CD1) and Trp(G8) for *Mt*. trHbO [58] and Tyr(B10) as well as Gln(E11) for *Mt*. trHbN. The homology model indicates that as for *Bs*. trHbO, *Rd*. trHb possesses a mixture of the conserved residues of group I and group II trHb of *M. tuberculosis* that are involved in the stabilization of the ligands. The only significant difference being His38, next to position CD1, that is present in trHbs from *Roseobacters* but absent in other trHbs. His38 in the CD region has not yet been reported and its role in ligand stabilization remains to be determined.

### Resonance Raman spectra

To gain information about the active site of *Rd*. trHb, resonance Raman spectra were obtained. The high-frequency region of the resonance Raman spectra of heme proteins comprises several in-plane vibrational modes of the porphyrin that are sensitive to the oxidation, coordination and spin-state of the heme-iron [59,60]. In the low-frequency region, in-plane and out-of-plane vibrational modes of the porphyrin macrocycle contribute to the spectra, in addition to the modes associated with the axial ligands of the heme-iron and the heme substituents (i.e. vinyl and propionate groups) [61].

**Fig 6:**
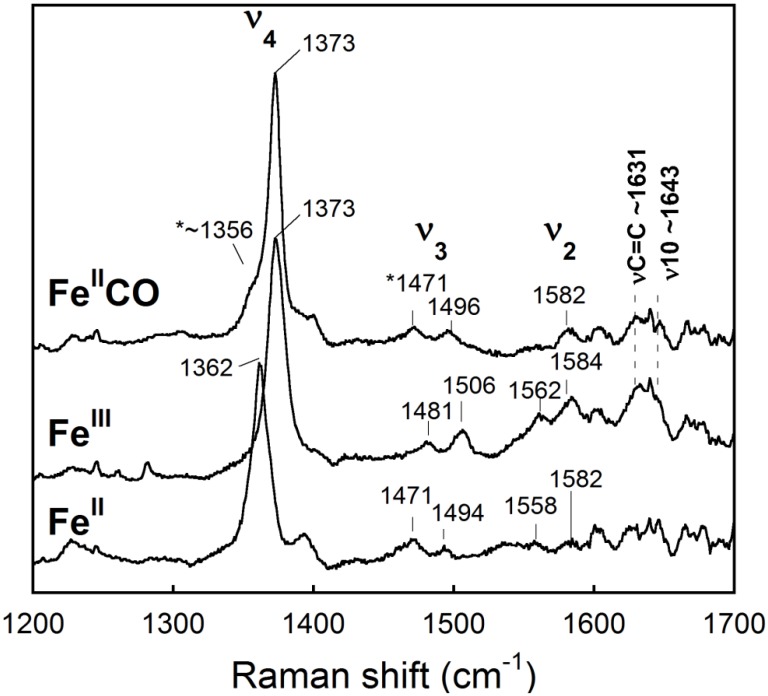
Resonance Raman spectra of *Rd*. trHb in the high frequency region. The spectra of the ferric (Fe^III^), reduced/ferrous (Fe^II^) and CO-bound complex were obtained with an excitation wavelength of 413.1 nm. The stars denote the ν_4_ and ν_3_ lines of some reduced, CO-free form of the protein present at low concentration because of laser-induced CO photo-dissociation.

Fig. 6 shows the high-frequency region of resonance Raman spectrum of *Rd*. trHb. The ν_4_ line at 1373 cm^-1^ is consistent with the heme being in the oxidized state. Two sets of ν_3_ and ν_2_ lines indicated the presence of a 6-coordinate high-spin heme (ν_3_ at 1481 cm^-1^ and ν_2_ at 1562 cm^-1^) and a 6-coordinate low-spin heme (ν_3_ at 1506 cm^-1^, ν_2_ at 1584 cm^-1^ and ν_10_ at ~1643 cm^-1^) (Table 2). The C = C stretching mode of the vinyl groups was detected at ~1631 cm^-1^. The ν_3_ and ν_2_ frequencies of both the high-spin and low-spin states are similar to those of ferric *Mt*. trHbO [52]. In the latter, the 6-coordinate high-spin signal comes from water and the 6-coordinate low-spin signal from a hydroxide ion bound to the heme. However, in contrast to *Mt*. trHbO, which shows a clear transition of low-spin to high-spin (or high-spin to low-spin) as the pH decreases (or increases) [55], only small spectral changes were detected in our pH titration (S2 Fig.). The Soret band was red-shifted from 412 nm to 416 nm as the pH was increased from 6 to 12 whereas the changes in the Q bands were rather small. *Rd*. trHb stayed mostly low-spin even at pH 6, which is clearly different from *Mt*. trHbO. It seems highly unlikely that a hydroxide ion would stay bound heme at pH 6. Rather, an amino acid is most likely the axial ligand at pH 6–7. As the pH increased, this amino acid may remain ligated or a transition to the hydroxide ion bound complex may take place. In either case, the optical spectra may only display small or no differences. Based on a structural model of *Rd*. trHb, potential amino acids that could coordinate the ferric heme-iron are Tyr22(B10) and Gln51(E11). Tyr(B10) was identified as the sixth ligand to the heme of ferric trHb of *Chlamydomonas eugametos*, a group I trHb [62]. To our knowledge, there is no precedent for Gln coordination to heme in trHbs.

**Table 2:** Summary of the heme skeletal modes of *Rd*. trHb in cm^-1^.

Complex	Coordination	ν_4_	ν_3_	ν_2_	ν_C = C_	ν_10_
Fe^III^-heme	6C HS	1373	1481	1562	-	-
	6C LS	1373	1506	1584	~1631	~1643
Fe^II^-heme	5C HS	1362	1471	1558	-	-
	6C LS		1494	1582	-	-
Fe^II^-heme-CO	6C LS	1373	1496	1582	~1631	~1643

-, not determined

We also investigated the high-frequency and low-frequency regions of Raman spectra of the ferrous state. Fig. 6 shows that the heme of the ferrous form contains some 5-coordinate high-spin heme with a strong ν_3_ line at 1471 cm^-1^, along with some 6-coordinate low-spin heme with ν_3_ at 1494 cm^-1^. The low-spin signal may come from an amino acid coordinating the reduced heme, presumably the same amino acid that coordinates the heme in the ferric state. It is interesting to note that for most trHbs characterized so far, the heme was found to be in the 5-coordinate state [63]. *Rd*. trHb with a mixture of 5- and 6-coordinate states appears more similar to the trHbs of photosynthetic organisms; namely *Synechocystis* trHb, which contains a 6-coordinate heme having a histidine as the sixth axial ligand [25], and *Chlamydomonas* trHb, which contains mostly a 5-coordinate heme at pH 7.5 that becomes 6-coordinate at higher pH [64].

**Fig 7:**
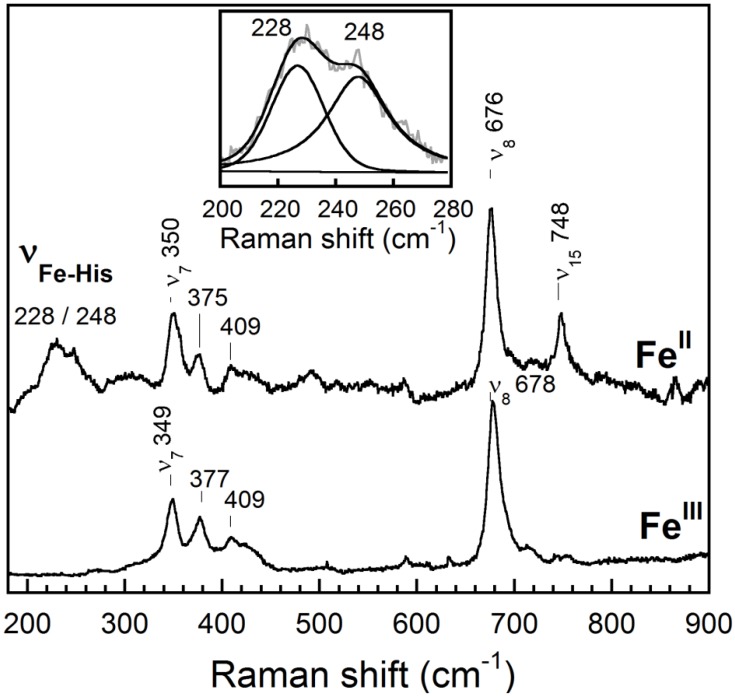
Resonance Raman spectra of ferric and ferrous *Rd*. trHb in the low-frequency region. The spectrum of the ferric state was obtained with an excitation wavelength of 413.1 nm while that of the reduced protein was obtained with an excitation wavelength of 441.6 nm. The inset shows the de-convolution in the 200–280 cm^-1^ range of the ferrous spectrum where two lines of similar area under the curve and of similar spectral bandwidth (24–25 cm^-1^) could be fitted. Several heme modes are identified on the figure. The lines at 375/377 and 409 cm^-1^ correspond to a bending mode of the propionate and of the vinyl groups, respectively. The resonance Raman spectrum of the ferrous protein obtained with an excitation wavelength of 413.1 nm, which preferentially enhances the signal from the 6 coordinate state, was almost featureless in the 200–250 nm region (not shown).

**Fig 8:**
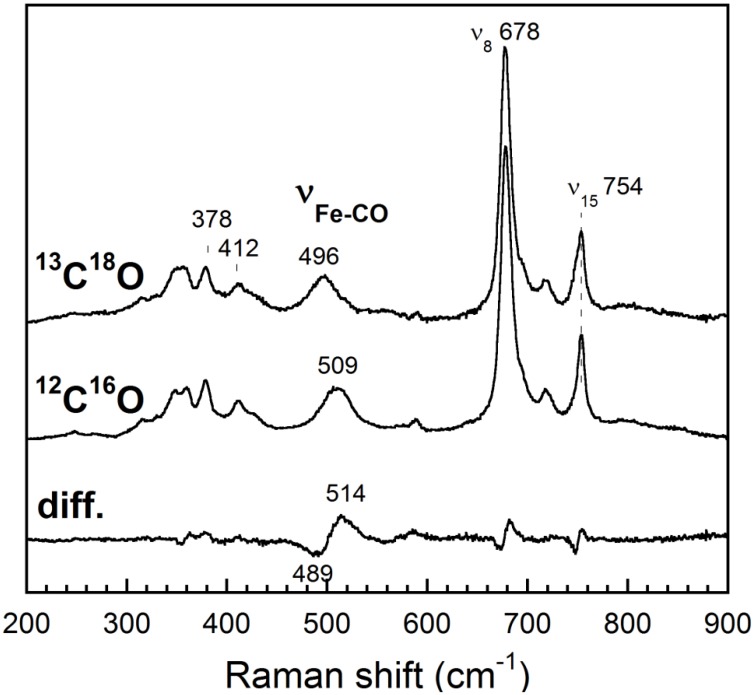
Resonance Raman spectra of *Rd*. trHb Fe(II)CO complex in the low-frequency region. An excitation wavelength of 413.1 nm was used. The difference spectrum (diff) is the spectrum obtained by subtracting the ^13^C^18^O spectrum from the ^12^C^16^O spectrum. The Fe-CO stretching mode (ν_Fe-CO_) and other heme modes are identified. The lines at 378 and 412 cm^-1^ correspond to a bending mode of the propionate and of the vinyl groups, respectively.

The low-frequency region was investigated using an excitation wavelength of 442 nm to enhance the signal from the 5-coordinate state and to identify the Fe-His stretching mode. In contrast to most reported Hbs, which contain one Fe-His stretching mode (ν_Fe-His_) in the 200–220 cm^-1^ region, *Rd*. trHb exhibits two Fe-His stretching modes at 228 and 248 cm^-1^, respectively (Fig. 7). We considered the possibility that the line at 248 cm^-1^ originated from the ν_9_ mode of the porphyrin, but this mode would be expected to be present in the ferric (Fig. 7) and Fe(II)-CO (Fig. 8) spectra, and these are featureless in this region. We thus conclude that the 248 cm^-1^ line more likely originates from a Fe-His mode that is known to be specifically enhanced from the 5-coordinate state of heme proteins and is probed here with an excitation wavelength at 442 nm. The Fe-His stretching mode at 248 cm^-1^ appears as a shoulder to the 228 cm^-1^ line but the fit of the spectrum in the 200–280 cm^-1^ region revealed that the area under the curve of the 248 cm^-1^ line was similar to that of the 228 cm^-1^ line, indicating that both Fe-His stretching modes are almost equally populated (Inset in Fig. 7). The frequency of the Fe-His stretching mode at 228 cm^-1^ is similar to that of *Mt*. trHbO (226 cm^-1^) and is accordingly assigned to a neutral histidine forming a favorable interaction with the heme-iron [52,63]. In contrast, the much higher frequency of the stretching mode at 248 cm^-1^ is similar to that of peroxidases where the proximal histidine is partially deprotonated by a strong hydrogen bond with a nearby aspartate/glutamate residue (i.e. an Fe-His-Asp/Glu triad) (Table 3) [65]. The flavohemoglobins and some bacterial single domain Hbs, such as Cgb of *Campylobacter jejuni*, also display an Fe-His stretching mode with such a high-frequency [66,67,68]. In *C. jejuni* Cgb, the histidine is part of an Fe-His-Glu triad (with Glu at the position H23). The catalytic triad is suggested to facilitate activation of the O-O bond of peroxide-bound peroxidases [69], which is highly relevant to the peroxidase activity we report for *Rd*. trHb (*vide infra*) and/or NO detoxification activities discussed further below. From the sequence alignment (Fig. 3), we note that *Rd*. trHb has an Asp (Asp129) at the position H23. However, the homology model of *Rd*. trHb (Fig. 5A) cannot be used to evaluate if this Asp may be in a position to form a strong hydrogen bond with the proximal His at F8 since it does not extend further than the position H19. Nevertheless, this Asp is notably conserved in trHbs of *Roseobacters* (Fig. 3), suggesting a functional role in these organisms. We also note that the C-terminus of *Rd*. trHb is more than 20 amino acids longer than trHbs of known structures and that this sequence is rich in Asp/Glu residues. This region may be another source for the amino acid involved in a Fe-His78-Asp/Glu catalytic triad. Altogether, the resonance Raman spectrum in the low-frequency region confirms iron coordination to a histidine in *Rd*. trHb and suggests a possible catalytic triad. The simple scenario is that the proximal histidine is His78 at F8, i.e. the same histidine as *Mt*. trHbO and *Bs*. trHbO, as suggested by the structural model (Fig. 5A). The low-frequency regions also displays lines at 350 and 676 cm^-1^ that are assigned to the ν_7_ and ν_8_ modes of the porphyrin ring [70], a line at 375 cm^-1^ that is assigned to a bending mode of a heme propionate, δ(C_β_C_c_C_d_), and a line at 409 cm^-1^ that is assigned to a bending mode of a vinyl group, δ(C_β_C_a_C_b_) [71] (Table 2 and Fig. 7). The protein environment surrounding the propionate and vinyl groups did not change significantly upon heme reduction as the frequencies of the δ(C_β_C_c_C_d_) and δ(C_β_C_a_C_b_) modes remained nearly the same in the reduced state compared to the ferric state.

**Table 3:** Summary of resonance Raman data of *Rd*. trHb, *Mt*. trHbO and *Bs*. trHbO in cm^-1^.

TrHbs	ν_Fe-His_	ν_Fe-CO_	Ref.
*Rd*. trHb	228, 248	509	This work
*Mt*. trHbO (wild-type)	226	525	[52]
*Mt*. trHbO (W(G8)F)	226	497, 514	[55]
*Mt*. trHbO (Y(CD1)F)	226	515	[52]
*Mt*. trHbO (W(G8)F—Y(CD1)F)	226	497	[55]
*Bs*. trHbO (wild-type)	-	520, 545	[56]
*Bs*. trHbO (W(G8)L)	-	489, 524	[56]

-, not determined

Fig. 6 shows that the Fe(II)CO complex of *Rd*. trHb is 6-coordinate and low-spin with ν_3_ at 1496 cm^-1^ and ν_2_ at 1582 cm^-1^. The relatively high-frequency of the oxidation state marker ν_4_ at 1373 cm^-1^ is typical of Fe(II)CO complexes of heme proteins and is indicative of π-back-bonding from the reduced iron to CO. Even at relatively low power (less than 2 mW), a small amount of photo-dissociation of CO occurred as revealed by the detection of the ν_4_ (~1356 cm^-1^) and ν_3_ (1471 cm^-1^) lines of a 5-coordinate reduced state (Fe^II^). These band increased in intensity at higher laser power (13 mW) and decreased at lower intensity (0.5 mW) indicating that this process was reversible (data not shown). The frequency of the ν_4_ line of the photo-dissociated reduced state (1356 cm^-1^) differed somewhat from that of the equilibrium reduced state (1362 cm^-1^) suggesting that only the 5-coordinate state may be present if the sixth ligand to the 6-coordinate state observed in the equilibrium sample had not formed yet.

Isotope substitution was used to identify the modes of heme-bound CO in the low-frequency region of the resonance Raman spectra of the Fe(II)CO complex of *Rd*. trHb (Fig. 8). The Fe-CO stretching mode (ν_Fe-CO_) was identified at 509 cm^-1^ (496 cm^-1^ with ^13^C^18^O). The frequency of the ν_Fe-CO_ mode remained the same (509 cm^-1^) for spectra recorded at 0.5 mW (not shown) and 2 mW laser power (Fig. 8). The frequency of the Fe-CO stretching mode of *Rd*. trHb is lower than that of wild-type *Mt*. trHbO (525 cm^-1^) [52], where CO interacts with the side-chain of two amino acids (Trp(G8) and Tyr(CD1)), and is more similar to its Trp(G8)Phe mutant (with ν_Fe-CO_ at 514 cm^-1^) and Tyr(CD1)Phe mutant (with ν_Fe-CO_ at 515 cm^-1^), where CO is interacting with a single amino acid residue [52,55] (Table 3). The Fe-CO stretching frequency of *Rd*. trHb is also notably much lower than those of the two conformers observed for *Bs*. trHbO (520 and 545 cm^-1^), although both proteins share common polar groups in the heme pocket (Tyr(B10), Thr(E7), Gln(E11) and Trp(G8)) [42,56] (Table 3). Trp(G8) was shown to be the a key player for ligand stabilization in trHbO of *B. subtilis* along with Tyr(B10) [56]. When CO is not experiencing any interaction with a nearby group, the ν_Fe_-_CO_ frequency is found near 495 cm^-1^, such as with the Trp(G8)Phe/Tyr(CD1)Phe double mutant of *Mt*. trHbO (497 cm^-1^) [55] and one conformer of the Trp(G8)Leu mutant of *Bs*. trHbO (489 cm^-1^) (Table 3) [56]. So the relatively high-frequency of ν_Fe-CO_ of *Rd*. trHb indicates that an amino acid residue interacts with the heme-bound CO but that this interaction is weaker than in *Mt*. trHbO and *Bs*. trHbO. Contrary to the latter, only one Fe-CO stretching mode was observed for *Rd*. trHb providing no evidence that more than one conformer is present. The multiple sequence alignment and the structural model (Fig. 5) indicate that a number of polar residues are present in the heme pocket, such as Trp91 at G8, Tyr22 at B10, Gln51 at E11 and His38 next to CD1, which is conserved in putative trHbs of *Roseobacters* (Fig. 3). These will be examined for their roles in ligand binding in our future studies. The C-O stretching mode could not be detected because the sample was highly fluorescent in the 1500–2200 cm^-1^ region (not shown).

### Gene expression of *Rd*. trHb, peroxidase reaction and implication of potential function(s)

**Fig 9:**
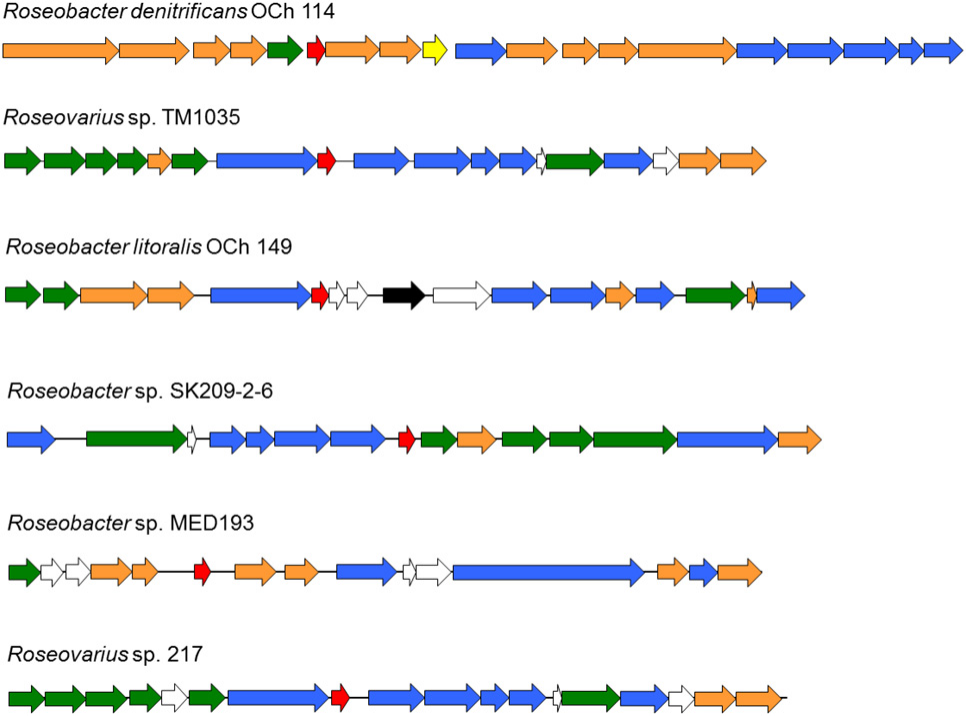
Genomic organization of the *glbO* gene in *Roseobacters*. The *glbO* gene encoding trHb is shown in **red**, genes encoding proteins for ammonium transfer are shown in **blue**, genes encoding proteins involved in nitrate/nitrite metabolism in **yellow**, genes encoding transporters and permeases in **green**, genes encoding transposases in **black**, genes encoding proteins with other functions in **orange** and genes encoding putative proteins of unknown functions in **white**.

The genomic sequence of *Roseobacters* revealed that the *glbO* gene (encoding trHb) is either adjacent or a part of a gene cluster encoding proteins responsible for nitrogen/ammonium transfer and transport, such as glutamate synthase, glutamine synthetase, (glutamine) amidotransferases, and ammonium/amino acid transporters and permeases (Fig. 9). Glutamate is a key metabolite connecting the central carbon and nitrogen metabolism and thus is involved in reducing or inducing oxidative stress [72,73]. Acquisition of glutamate via the glutamate transporter was suggested to be important for *Francisella tularensis* for protection against oxidative stress [74]. Further, glutamine synthetase is also known to be sensitive to oxidative stress [75]. Thus the genomic environment suggests that *Rd*. trHb may be involved in nitrogen metabolism and oxidative stress response in *Roseobacters*.

In addition to binding molecular oxygen, CO and nitric oxide (NO), trHbs are known to undergo allosteric regulation like Hbs in Metazoan [76] and also function as biological catalysts (enzymes) [17]. In terms of potential function(s) of *Rd*. trHb, one of the *Campylobacter jejuni* trHbs was suggested to function as a peroxidase [77] and *Mt*. trHbO, as well as its homologous trHb protein from *Thermobifida fusca* (*Tf*. trHbO), have been shown to function as peroxidases that reduce hydrogen peroxide while oxidizing an organic substrate [78,79]. Tyr at position CD1 of *Mt*. trHbO is involved in radical propagation in the protein following exposure to hydrogen peroxide and two surface Tyr residues (Y55 and Y115) have been shown to form radicals, which led to the formation of protein dimers [78]. In contrast, *Rd*. trHb has Phe at CD1, like in all full-length Hbs (i.e. those with a 3-on-3 alpha-helical fold) and many trHbs, and the two surface Tyr residues of *Mt*. HbO are not conserved in *Rd*. trHb, which has Phe and Thr at those sites. Thus any peroxidase activity by *Rd*. trHb would neither propagate a radical through Tyr at CD1 nor involve the surface Tyr residues identified in *Mt*. trHb. However, *Rd*. trHb could still oxidize substrates by radical propagation through other residue(s) or from the compound I intermediate (ferryl species with an associated radical) as in most peroxidases.

**Fig 10:**
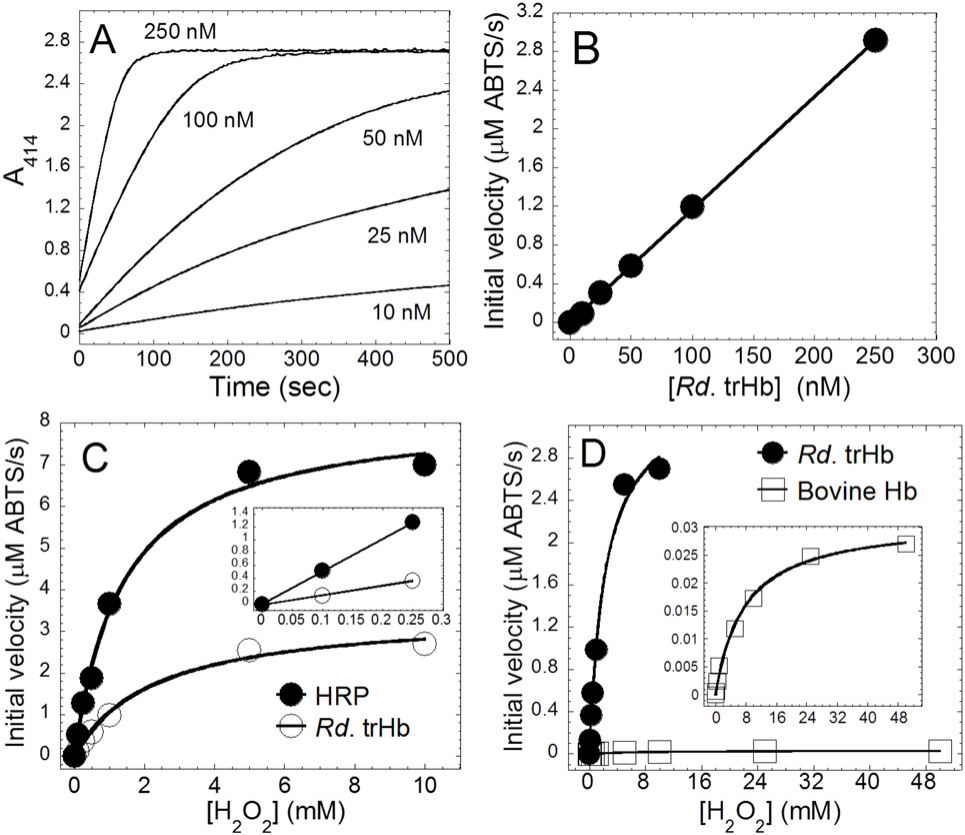
Steady-state kinetics of the peroxidase reaction catalyzed by *Rd*. trHb, bovine Hb and horseradish peroxidase (HRP). The substrates H_2_O_2_ (0.5 mM) and ABTS (0.5 mM) were mixed with various concentrations of *Rd*. trHb (10–250 nM) in 20 mM Tris-HCl at pH 8.0 (A). The oxidation of ABTS catalyzed by *Rd*. trHb was monitored by the increase of absorbance at 414 nm (A_414_). The initial velocities (μM ABTS/s) showed a linear relationship with respect to versus the concentration of *Rd*. trHb (B). Plot of the initial velocities versus the concentration of H_2_O_2_, where 13.5 nM HRP or 50 nM *Rd*. trHb was mixed with various concentrations of H_2_O_2_/ABTS in 20 mM Tris-HCl (pH 8.0). The inset shows the linear fit of the HRP and *Rd*. trHb data up to 0.25 mM H_2_O_2_ (C). Plot of the initial velocities versus the concentration of H_2_O_2_, where 50 nM *Rd*. trHb or 77.5 nM bovine Hb was mixed with various concentrations of H_2_O_2_/ABTS in 20 mM Tris-HCl (pH 8.0). The inset shows the rescaled plot of bovine Hb (D).

To examine the possible catalytic activity of *Rd*. trHb, we assayed the peroxidase activity using H_2_O_2_ and ABTS as substrates. The reaction was monitored by the increase of absorbance at 414 nm (A_414_) caused by the oxidation of ABTS. No increase of A_414_ was detected without the addition of *Rd*. trHb. Fig. 10A shows the increase of absorbance at 414 nm with different concentrations of *Rd*. trHb (0 to 250 nM), and Fig. 10B illustrates the linear relationship of the initial velocities (μM ABTS/s) versus the concentration of *Rd*. trHb (0 to 250 nM). The straight line indicates that with ABTS and H_2_O_2_ both at 0.5 mM concentration, *Rd*. trHb did not show apparent enzymatic inactivation.

To investigate the steady-state kinetics of the peroxidase activity catalyzed by *Rd*. trHb, we estimated the initial velocities of ABTS oxidation by *Rd*. trHb at various concentrations of [H_2_O_2_] and compared the results with those of the well characterized HRP (Fig. 10C). When 50 nM *Rd*. trHb or 13.5 nM HRP were employed, *Rd*. trHb displayed a similar *K*_m,app_ (apparent *K*_m_) and approximately one order of magnitude lower *k*_cat,app_ (apparent *k*_cat_) and *k*_cat,app_/*K*_m,app_ values compared to HRP (Table 4). The peroxidase activity of *Rd*. trHb (50 nM) was also much higher than that of bovine Hb (77.5 nM) (Fig. 10D and Table 4), which is a heme protein with a very low but measurable peroxidase activity [80]. Unlike *Rd*. trHb, the trHbO from *M. tuberculosis* and *T. fusca* did not show saturation kinetics with respect to the concentration of H_2_O_2_ when ABTS was the organic substrate [78,79]. To compare *Rd*. trHb with these trHbOs, we used the slope of the initial rates versus concentration of H_2_O_2_ with data points up to 0.25 mM H_2_O_2_. This slope indicates the apparent first-order rate dependence of the reaction with respect of H_2_O_2_ as described for *Mt*. and *Tf*. trHbO [78,79]. This analysis of the *Rd*. trHb data gives a very similar value (1.5 x 10^3^ M^-1^s^-1^) (Inset in Fig. 10C) compared to those determined for *Mt*. trHbO (1.4 x 10^3^ M^-1^s^-1^) and *Tf*. trHbO (1.9 x 10^3^ M^-1^s^-1^). This slope is also a measure of the apparent *k*_cat_/*K*_m_ when the concentration of the variable substrate is small relative to *K*_m_ which reduces the Michaelis-Menten equation to *V* = *V*_max_/*K*_m_ or *k* = *k*_cat_/*K*_m_ when the initial rates are divided by the concentration of enzyme. Such an analysis gives a *k*_cat_/*K*_m_ ratio of 3.0 x 10^4^ M^-1^s^-1^ which is consistent with the *k*_*cat*_/*K*_*m*_ ratio calculated from the apparent *k*_cat_ and *K*_m_ values (Table 4). Thus, our results show overall that *Rd*. trHb has a very significant peroxidase activity similar to that of *Mt*. and *Tf*. trHbO although it differs from the latter in showing saturation kinetics with respect to H_2_O_2_ when ABTS is the organic substrate.

**Table 4:** The steady-state kinetic parameters of the peroxidase reaction catalyzed by HRP, *Rd*. trHb and bovine Hb at pH 8.

Kinetic parameters	HRP	*Rd*. trHb	Bovine Hb
*V*_max,app_ (μM•s^-1^)	8.3 ± 0.3	3.4 ± 0.2	0.03 ± 0.001
*k*_cat,app_ (s^-1^)	615 ± 31	68 ± 6	0.39 ± 0.02
*K*_m,app_ (mM)	1.4 ± 0.2	2.3 ± 0.3	7.5 ± 0.9
*k*_cat,app_/*K*_m,app_ (M^-1^•s^-1^)	(4.4 ± 0.4) x 10^5^	(3.1 ± 0.2) x 10^4^	52 ± 4

**Fig 11:**
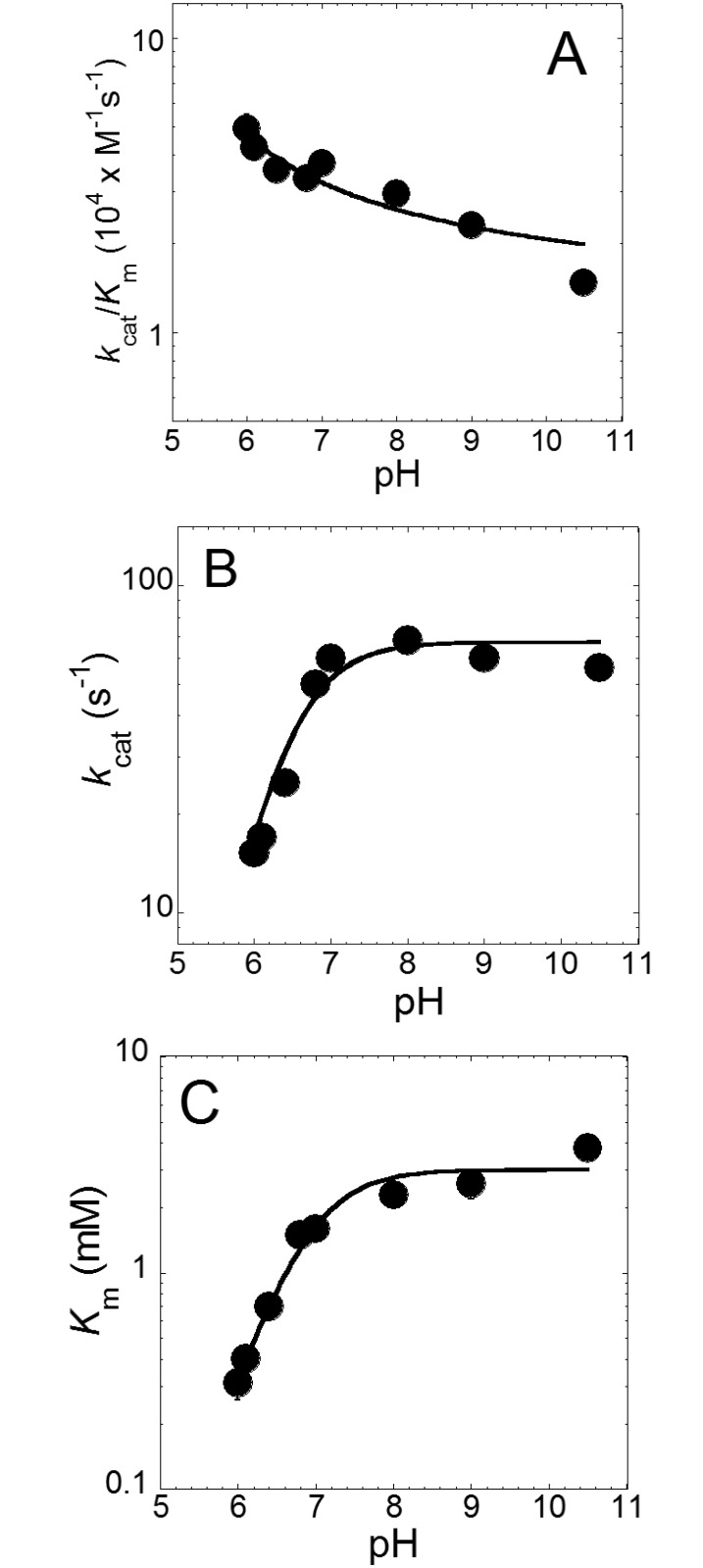
Peroxidase reaction catalyzed by *Rd*. trHb at different pHs. The pH profiles of *k*_cat,app_/*K*_m,app_ (A), *k*_cat_,_app_ (B), *K*_m,app_ (C) are shown.

To enhance our understanding on the peroxidase reaction catalyzed by *Rd*. trHb, we performed steady-state kinetic measurements with H_2_O_2_/ABTS in buffers of different pHs (pH 6 to 10.5) (Table 5). The peroxidase reaction catalyzed by *Rd*. trHb showed similar catalytic efficiencies in the pH range investigated, with *k*_cat,app_/*K*_m,app_ values within a 5-fold range (Fig. 11A). The *k*_cat,app_/*K*_m,app_ ratios were slightly greater at lower pH than at higher pH (Fig. 11A). Nevertheless, compared with the reactions catalyzed at neutral to mild-alkaline pH (pH 7 and 8), at pH 6, both the *K*_m,app_ and the turnover rate were smaller (Fig. 11B and 11C). The smaller *K*_m,app_ value suggests that H_2_O_2_ apparently binds more tightly to the heme at low pH despite being harder to deprotonate. It should be cautioned that the steady-state kinetic parameters reported in this paper were measured from the oxidation of ABTS, so possible pH-dependent effect of ABTS binding to the active site of *Rd*. trHb cannot be ruled out. A slower catalytic turnover may be correlated with a lower fraction of the proximal histidine expected to be deprotonated at low pH (i.e. having an imidazolate-character). Data fitted with equations 1 and 2 described in Materials and Methods indicate that all pH profiles yield one p*K*_a_ value: 6.5 ± 0.2 (*k*_cat,app_), 7.0 ± 0.3 (*K*_m,app_) and 6.8 ± 0.2 (*k*_cat,app_/*K*_m,app_) (Table 5). Thus, a p*K*_a_ of ~6.8, which is close to the p*K*_a_ of the side chain of histidine free form (~6.0), is observed in *k*_cat,app_, *K*_m,app_ and *k*_cat,app_/*K*_m,app_. If the p*K*_a_ value of ~6.8 represents the p*K*_a_ of the proximal histidine of *Rd*. trHb, it could explain that a significant fraction of the protein displayed a proximal histidine with imidazolate character as detected from the resonance Raman spectrum collected in a buffer at pH 8.0 (Fig. 7).

**Table 5:** The steady-state kinetic parameters of the peroxidase reaction catalyzed by *Rd*. trHb at various pHs.

	*V*_max,app_ (μM•s^-1^)	*k*_cat,app_ (s^-1^)	*K*_m,app_ (mM)	*k*_cat,app_/*K*_m,app_ (10^4^ x M^-1^•s^-1^)
pH 6.0	0.76 ± 0.03	15.2 ± 0.6	0.31 ± 0.05	5.0 ± 0.6
pH 6.2	0.85 ± 0.04	17 ± 0.8	0.4 ± 0.06	4.5 ± 0.4
pH 6.5	1.25 ± 0.08	25 ± 1.4	0.7 ± 0.1	3.6 ± 0.3
pH 6.8	2.5 ± 0.1	50 ± 2	1.5 ± 0.1	3.3 ± 0.1
pH 7.0	3.0 ± 0.1	60 ± 2	1.6 ± 0.1	3.8 ± 0.1
pH 8.0	3.4 ± 0.2	68 ± 6	2.3 ± 0.3	3.1 ± 0.2
pH 9.0	3.0 ± 0.2	60 ± 4	2.6 ± 0.4	2.3 ± 0.2
pH 10.5	2.8 ± 0.1	56 ± 2	3.8 ± 0.4	1.5 ± 0.1
p*K*_a_		6.5 ± 0.2	7.0 ± 0.3	6.8 ± 0.4

**Fig 12:**
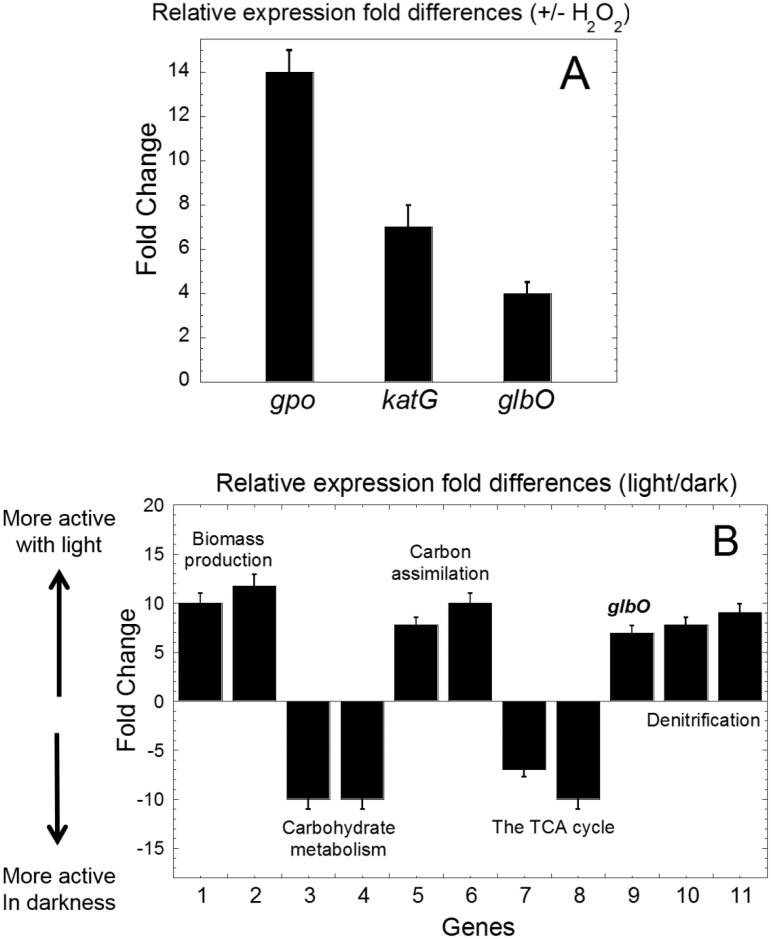
Transcript level profiles of some *R. denitrificans genes*. Probe of the transcript expression levels of *gpo* (glutathione peroxidase), *katG* (bi-functional catalase-peroxidase) and *glbO* (truncated hemoglobin) in cultures grown chemotrophically in a minimal medium supplied with 10 mM acetate and with or without 1 mM H_2_O_2_ (A), and probe of the transcript expression level of genes involved in carbon and nitrogen metabolism in cultures grown in a minimal medium supplied with 10 mM glucose and subjected to cycles of 12-h light followed by 12-h dark periods (B): **1**. RD1_2870 (phosphoglucomutase); **2**. RD1_2720 (glucose-6-phosphate isomerase); **3**. RD1_2879 (6-phosphogluconate dehydrase); **4**. RD1_2878 (KDPG aldolase); **5**. RD1_3376 (pyruvate carboxylase); **6**. RD1_0421 (malic enzyme); **7**. RD1_1609 (2-ketoglutarate dehydrogenase); **8**. RD1_2204 (isocitrate dehydrogenase); **9**. RD1_4240 (*glbO*, truncated hemoglobin); **10**. RD1_1561 (NO reductase); **11**. RD1_1562 (NO reductase).

Although *Rd*. trHb displays clear differences of its active site compared to *Mt*. and *Tf*. trHbO, it shares with the latter a significant peroxidase activity (see above), and thus the ability to oxidize an organic substrate using hydrogen peroxide [78]. Meanwhile, bi-functional catalase/peroxidase (encoded by the *katG* gene), which has been detected in several *Roseobacters* [81,82,83], may be related to oxidation of secondary metabolites and production of biofilms [84,85,86,87,88], as well as glutathione peroxidase (encoded by the *gpo* gene), which is known to detoxify ROS in aerobic organisms [8,37]. Herein, we examined the possible physiological role of *Rd*. trHb *in vivo* using gene expression experiments (Fig. 12A). *R. denitrificans* was grown in minimal medium containing acetate with or without H_2_O_2_ (1 mM) and the transcript levels of *glbO* (RD1_4240), *katG* (RD1_2195) and *gpo* (RD1_0599) were examined during the exponential growth phase. Significant up-regulation of the *gpo* and *katG* genes, along with moderate increment of *glbO*, were observed in the cultures exposed to H_2_O_2_, compared with cultures without H_2_O_2_, implying potential detoxification of reactive oxygen via *Rd*. trHb *in vivo*.

Different globins are known to possess a NO dioxygenase activity that converts NO to harmless nitrate from the reaction of the oxygenated state of these protein with NO [57,89,90,91]. *M. tuberculosis* was proposed to use *Mt*. trHbN to detoxify NO produced by macrophages [92], whereas *Mt*. trHbO was shown to have a 1,200-fold reduced NO dioxygenase activity compared with *Mt*. trHbN making it unlikely that it is involved in such a reaction [58]. It was noted that proteins that possess a NO dioxygenase activity share the property of having more than one residue interacting with the heme bound ligand, such as carbon monoxide. For these proteins, a dynamic conversion of open and closed state was suggested from the detection of two Fe-CO stretching modes of the Fe(II)-CO complex [68]. The single Fe-CO stretching mode detected for *Rd*. trHb distinguishes this protein. However, the imidazolate character of the Fe-His stretching mode of flavohemoglobins and the single chain hemoglobin Cgb of *C. jejuni*, which is believed to favor their NO dioxygenase activity, is a feature shared by *Rd*. TrHb [68]. Interestingly, it was proposed that trHbO of *Mycobacterium lepreae* is involved in both H_2_O_2_ and NO scavenging [93]. For this protein, it was proposed that the reaction of the ferric protein with H_2_O_2_ would lead to the formation of a ferryl species that would then react with NO to produce nitrite. Such a reaction bypasses the need of a reductase to reduce the heme and to allow oxygen binding as occurs in flavohemoglobins as they carry out the NO dioxygenase reaction. The relatively efficient reaction with peroxide catalyzed by *Rd*. trHb discussed above may be relevant to such a NO detoxification reaction.

An enzyme with a NO detoxification activity, either through a NO dioxygenase reaction or through a reaction involving a ferryl heme and NO, would be helpful during aerobic denitrification in *R. denitrificans*. While aerobic denitrification is no longer recognized as an uncommon process in nature [94], *R. denitrificans* has not yet been shown to grow under strictly denitrification condition [10] and thus, it seems unlikely that it uses nitrate denitrification as an energy source. Since *R. denitrificans* cannot grow aerobically without the supply of nitrate (data not shown), the biological and physiological roles of its aerobic denitrification activity, in addition to consuming excess reducing equivalents to minimize the production of ROS [95], remains to be understood. Here, we used QRT-PCR to monitor the expression level of gene transcripts from cultures grown in the presence of glucose in day-night growth cycles. Fig. 12B shows that *glbO* and genes involved in denitrification, biomass production and carbon assimilation are stimulated by light, whereas genes involved in carbohydrate metabolism and the TCA cycle are repressed. These observations are in agreement with the fact that *Roseobacters* perform photosynthesis during phototrophic growth and obtain energy from the oxidation of organic carbons via aerobic respiration during chemotrophic growth [96]. Moreover, up-regulation of the NO reductase gene indicated from our data, along with the known higher cellular levels of the nitrite reductase (cytochrome *cd*_1_ complex) [11] and activation of the aerobic denitrification process by light [9], suggest that more NO molecules are generated during phototrophic growth.

NO produced by aerobic denitrification may function as a signal transducer to inhibit the cytochrome *c* oxidase activity and thus could down-regulate aerobic respiration, as reported in mitochondria [97]. NO is also known to inactivate aconitase of the TCA cycle and 6-phospho-gluconate dehydratase of the Entner-Doudoroff (ED) pathway [98]. The latter is the pathway by which *R. denitrificans* breaks down sugars to produce pyruvate molecules and synthesizes ATP, NADH and NADPH to preserve energy [37]. Alternatively, active carbon assimilation and biomass production [99], which consume reducing equivalents (NADPH) produced during aerobic photosynthesis, would be stimulated to minimize the production of ROS. NADPH is known to be one of the major source for the production of ROS [100] and *Roseobacters* have been suggested recently to be the major source of ROS in ocean [5]. ROS are not desirable during aerobic photosynthesis. They also contribute to the production of RNS and toxic peroxynitrite via reaction with NO. It is possible that *R. denitrificans* produces more trHb to convert excess NO and O_2_ to nitrate/nitrite during phototrophic growth. A similar mechanism was also suggested recently to link the activity of THB1, which is a trHb from *Chlamydomonas reinhardtii* that exhibits NO dioxygenase activity, to nitrogen assimilation in this organism [101]. This hypothesis will be verified in future studies.

## CONCLUSIONS

Hemoglobins are known to bind various gas molecules and some of them also possess enzymatic activities. Analysis using bioinformatic tools suggest that putative globin-like proteins belonging to the truncated globin family are present in *Roseobacters*. In this paper, we characterized the recombinant trHb from *R. denitrificans* using functional, spectral and modeling studies. The sequence alignment suggests that *Rd*. trHb belongs to group II trHbs and contains distal amino acids known to participate in ligand stabilization in this group of heme proteins. Resonance Raman spectra reveal a heme pocket structure consistent with the very significant peroxidase activity measured by steady-state kinetics with *Rd*. trHb. Together, these properties suggest a role for *Rd*. trHb in mechanisms of defense against reactive oxygen and/or nitrogen species or in redox biochemical reactions that remain to be explored further. Gene expression analyses also suggest that *Rd*. trHb contributes the detoxification of reactive oxygen and nitrogen species *in vivo*. Current genomic sequence data indicate that trHbs genes are widely distributed in the ecologically important marine *Roseobacters*. Some of the distinctive properties reported in this paper set the stage for further in-depth analyses of this interesting enzyme.

## Supporting Information

S1 Fig Superposition of the α-subunit of human Hb (shown in pink) (PDB ID 1GZX) with group I *Mycobacterium tuberculosis* trHb (*Mt*. trHbN) (shown in green) (PDB ID 1S56). (TIF)

S2 Fig UV-visible absorption spectra of *Rd*. trHb at various pHs. (TIF)

## References

[1] Beja O, Suzuki MT, Heidelberg JF, Nelson WC, Preston CM, et al (2002) Unsuspected diversity among marine aerobic anoxygenic phototrophs. Nature 415: 630–633. 11832943 10.1038/415630a

[2] Selje N, Simon M, Brinkhoff T (2004) A newly discovered *Roseobacter* cluster in temperate and polar oceans. Nature 427: 445–448. 14749832 10.1038/nature02272

[3] Wagner-Dobler I, Biebl H (2006) Environmental biology of the marine Roseobacter lineage. Annu Rev Microbiol 60: 255–280. 16719716 10.1146/annurev.micro.60.080805.142115

[4] Kolber ZS, Plumley FG, Lang AS, Beatty JT, Blankenship RE, et al (2001) Contribution of aerobic photoheterotrophic bacteria to the carbon cycle in the ocean. Science 292: 2492–2495. 11431568 10.1126/science.1059707

[5] Diaz JM, Hansel CM, Voelker BM, Mendes CM, Andeer PF, et al (2013) Widespread production of extracellular superoxide by heterotrophic bacteria. Science 340: 1223–1226. 10.1126/science.1237331 23641059

[6] Shiba T, Simidu U, Taga N (1979) Distribution of aerobic bacteria which contain bacteriochlorophyll *a* . Appl Environ Microbiol 38: 43–45. 16345414 10.1128/aem.38.1.43-45.1979 PMC243433

[7] Shiba T (1991) *Roseobacter litoralis* gen. nov., sp. nov. and *Roseobacter denitrificans* sp. nov., aerobic pink-pigmented bacteria which contain bacteriochlorophyll *a* . Syst Appl Microbial 14: 140–145.

[8] Swingley WD, Sadekar S, Mastrian SD, Matthies HJ, Hao J, et al (2007) The complete genome sequence of *Roseobacter denitrificans* reveals a mixotrophic rather than photosynthetic metabolism. J Bacteriol 189: 683–690. 17098896 10.1128/JB.01390-06 PMC1797316

[9] Doi M, Shioi Y (1991) Enhancement of denitrifying activity in cells of *Roseobacter denitrificans* grown aerobically in the light Plant Cell Physiol 32: 365–370.

[10] Shapleigh JP (2011) Oxygen control of nitrogen oxide respiration, focusing on -Proteobacteria. Biochem Soc Trans 39: 179–183. 10.1042/BST0390179 21265769

[11] Takamiya K, Satoh K, Okuyama T, Uchino R, Shioi Y, et al (1993) Increases in cellular levels of cytochrome cd1 in *Roseobacter denitrificans* upon irradiation with green light during aerobic growth Plant Cell Physiol 34: 985–990.

[12] Takamiya K, Shioi Y, Morita M, Arata H, Shimizu M, et al (1993) Some properties and occurrence of cytochrome c-552 in the aerobic photosynthetic bacterium *Roseobacter denitrificans* . Arch Microbiol 159: 51–56. 8381264 10.1007/BF00244264

[13] Garcia D, Richaud P, Breton J, Vermeglio A (1994) Structure and function of the tetraheme cytochrome associated to the reaction center of *Roseobacter denitrificans* . Biochimie 76: 666–673. 7893818 10.1016/0300-9084(94)90143-0

[14] Schwarze C, Carluccio AV, Venturoli G, Labahn A (2000) Photo-induced cyclic electron transfer involving cytochrome bc1 complex and reaction center in the obligate aerobic phototroph *Roseobacter denitrificans* . Eur J Biochem 267: 422–433. 10632712 10.1046/j.1432-1327.2000.01018.x

[15] Matsuda Y, Inamori K, Osaki T, Eguchi A, Watanabe A, et al (2002) Nitric oxide-reductase homologue that contains a copper atom and has cytochrome c-oxidase activity from an aerobic phototrophic bacterium *Roseobacter denitrificans* . J Biochem 131: 791–800. 12038974 10.1093/oxfordjournals.jbchem.a003167

[16] Kimura M, Ishii M, Igarashi Y, Arai H (2012) Identification of the genes encoding nitric oxide reductase in the aerobic photosynthetic bacterium *Roseobacter denitrificans* OCh114. Biosci Biotechnol Biochem 76: 1984–1986. 23047089 10.1271/bbb.120406

[17] Wittenberg JB, Bolognesi M, Wittenberg BA, Guertin M (2002) Truncated hemoglobins: a new family of hemoglobins widely distributed in bacteria, unicellular eukaryotes, and plants. J Biol Chem 277: 871–874. 11696555 10.1074/jbc.R100058200

[18] Nie X, Durnin DC, Igamberdiev AU, Hill RD (2006) Cytosolic calcium is involved in the regulation of *barley* hemoglobin gene expression. Planta 223: 542–549. 16177910 10.1007/s00425-005-0094-y

[19] Igamberdiev AU, Stoimenova M, Seregelyes C, Hill RD (2006) Class-1 hemoglobin and antioxidant metabolism in alfalfa roots. Planta 223: 1041–1046. 16284778 10.1007/s00425-005-0145-4

[20] Manac’h-Little N, Igamberdiev AU, Hill RD (2005) Hemoglobin expression affects ethylene production in maize cell cultures. Plant Physiol Biochem 43: 485–489. 15914016 10.1016/j.plaphy.2005.03.012

[21] Watts RA, Hunt PW, Hvitved AN, Hargrove MS, Peacock WJ, et al (2001) A hemoglobin from plants homologous to truncated hemoglobins of microorganisms. Proc Natl Acad Sci U S A 98: 10119–10124. 11526234 10.1073/pnas.191349198 PMC56925

[22] Falzone CJ, Christie Vu B, Scott NL, Lecomte JT (2002) The solution structure of the recombinant hemoglobin from the cyanobacterium *Synechocystis* sp. PCC 6803 in its hemichrome state. J Mol Biol 324: 1015–1029. 12470956 10.1016/s0022-2836(02)01093-8

[23] Scott NL, Falzone CJ, Vuletich DA, Zhao J, Bryant DA, et al (2002) Truncated hemoglobin from the cyanobacterium *Synechococcus* sp. PCC 7002: evidence for hexacoordination and covalent adduct formation in the ferric recombinant protein. Biochemistry 41: 6902–6910. 12033922 10.1021/bi025609m

[24] Hill DR, Belbin TJ, Thorsteinsson MV, Bassam D, Brass S, et al (1996) GlbN (cyanoglobin) is a peripheral membrane protein that is restricted to certain *Nostoc* spp. J Bacteriol 178: 6587–6598. 8932316 10.1128/jb.178.22.6587-6598.1996 PMC178546

[25] Couture M, Das TK, Savard PY, Ouellet Y, Wittenberg JB, et al (2000) Structural investigations of the hemoglobin of the cyanobacterium *Synechocystis* PCC6803 reveal a unique distal heme pocket. Eur J Biochem 267: 4770–4780. 10903511 10.1046/j.1432-1327.2000.01531.x

[26] Hvitved AN, Trent JT 3rd, Premer SA, Hargrove MS (2001) Ligand binding and hexacoordination in *Synechocystis* hemoglobin. J Biol Chem 276: 34714–34721. 11438545 10.1074/jbc.M105175200

[27] Hemschemeier A, Duner M, Casero D, Merchant SS, Winkler M, et al (2013) Hypoxic survival requires a 2-on-2 hemoglobin in a process involving nitric oxide. Proc Natl Acad Sci U S A 110: 10854–10859. 10.1073/pnas.1302592110 23754374 PMC3696821

[28] Couture M, Chamberland H, St-Pierre B, Lafontaine J, Guertin M (1994) Nuclear genes encoding chloroplast hemoglobins in the unicellular green alga *Chlamydomonas eugametos* . Mol Gen Genet 243: 185–197. 8177215 10.1007/BF00280316

[29] Pesce A, Couture M, Dewilde S, Guertin M, Yamauchi K, et al (2000) A novel two-over-two alpha-helical sandwich fold is characteristic of the truncated hemoglobin family. EMBO J 19: 2424–2434. 10835341 10.1093/emboj/19.11.2424 PMC212751

[30] Milani M, Pesce A, Ouellet Y, Ascenzi P, Guertin M, et al (2001) *Mycobacterium tuberculosis* hemoglobin N displays a protein tunnel suited for O2 diffusion to the heme. EMBO J 20: 3902–3909. 11483493 10.1093/emboj/20.15.3902 PMC149180

[31] Pesce A, Dewilde S, Kiger L, Milani M, Ascenzi P, et al (2001) Very high resolution structure of a trematode hemoglobin displaying a TyrB10-TyrE7 heme distal residue pair and high oxygen affinity. J Mol Biol 309: 1153–1164. 11399085 10.1006/jmbi.2001.4731

[32] Milani M, Pesce A, Ouellet Y, Dewilde S, Friedman J, et al (2004) Heme-ligand tunneling in group I truncated hemoglobins. J Biol Chem 279: 21520–21525. 15016811 10.1074/jbc.M401320200

[33] Milani M, Savard PY, Ouellet H, Ascenzi P, Guertin M, et al (2003) A TyrCD1/TrpG8 hydrogen bond network and a TyrB10TyrCD1 covalent link shape the heme distal site of *Mycobacterium tuberculosis* hemoglobin O. Proc Natl Acad Sci U S A 100: 5766–5771. 12719529 10.1073/pnas.1037676100 PMC156275

[34] Pesce A, Bolognesi M, Nardini M (2013) The diversity of 2/2 (truncated) globins. Adv Microb Physiol 63: 49–78. 10.1016/B978-0-12-407693-8.00002-9 24054794

[35] Vinogradov SN, Bailly X, Smith DR, Tinajero-Trejo M, Poole RK, et al (2013) Microbial eukaryote globins. Adv Microb Physiol 63: 391–446. 10.1016/B978-0-12-407693-8.00009-1 24054801

[36] Vinogradov SN, Moens L (2008) Diversity of globin function: enzymatic, transport, storage, and sensing. J Biol Chem 283: 8773–8777. 10.1074/jbc.R700029200 18211906

[37] Tang KH, Feng X, Tang YJ, Blankenship RE (2009) Carbohydrate metabolism and carbon fixation in *Roseobacter denitrificans* OCh114. PLoS One 4: e7233 10.1371/journal.pone.0007233 19794911 PMC2749216

[38] Tang KH, Harms A, Frey PA (2002) Identification of a novel pyridoxal 5′-phosphate binding site in adenosylcobalamin-dependent lysine 5,6-aminomutase from *Porphyromonas gingivalis* . Biochemistry 41: 8767–8776. 12093296 10.1021/bi020255k

[39] Tang KH, Wen J, Li X, Blankenship RE (2009) Role of the AcsF protein in *Chloroflexus aurantiacus* . J Bacteriol 191: 3580–3587. 10.1128/JB.00110-09 19346304 PMC2681904

[40] Childs RE, Bardsley WG (1975) The steady-state kinetics of peroxidase with 2,2′-azino-di-(3-ethyl-benzthiazoline-6-sulphonic acid) as chromogen. Biochem J 145: 93–103. 1191252 10.1042/bj1450093 PMC1165190

[41] Chartier FJ, Couture M (2004) Stability of the heme environment of the nitric oxide synthase from *Staphylococcus aureus* in the absence of pterin cofactor. Biophys J 87: 1939–1950. 15345570 10.1529/biophysj.104.042119 PMC1304597

[42] Giangiacomo L, Ilari A, Boffi A, Morea V, Chiancone E (2005) The truncated oxygen-avid hemoglobin from *Bacillus subtilis*: X-ray structure and ligand binding properties. J Biol Chem 280: 9192–9202. 15590662 10.1074/jbc.M407267200

[43] Reeder BJ, Hough MA (2014) The structure of a class 3 nonsymbiotic plant haemoglobin from *Arabidopsis thaliana* reveals a novel N-terminal helical extension. Acta Crystallogr D Biol Crystallogr 70: 1411–1418. 10.1107/S1399004714004878 24816109

[44] Pesce A, Nardini M, Labarre M, Richard C, Wittenberg JB, et al (2011) Structural characterization of a group II 2/2 hemoglobin from the plant pathogen Agrobacterium tumefaciens. Biochim Biophys Acta 1814: 810–816. 10.1016/j.bbapap.2010.11.001 21070893

[45] Edgar RC (2004) MUSCLE: multiple sequence alignment with high accuracy and high throughput. Nucleic Acids Res 32: 1792–1797. 15034147 10.1093/nar/gkh340 PMC390337

[46] Edgar RC (2004) MUSCLE: a multiple sequence alignment method with reduced time and space complexity. BMC Bioinformatics 5: 113 15318951 10.1186/1471-2105-5-113 PMC517706

[47] Molecular Operating Environment (MOE) CCGI, 1010 Sherbooke St. West, Suite #910, Montreal, QC, Canada, H3A 2R7, 2013.

[48] Chen VB, Arendall WB 3rd, Headd JJ, Keedy DA, Immormino RM, et al (2010) MolProbity: all-atom structure validation for macromolecular crystallography. Acta Crystallogr D Biol Crystallogr 66: 12–21. 10.1107/S0907444909042073 20057044 PMC2803126

[49] Tang KH, Yue H, Blankenship RE (2010) Energy metabolism of *Heliobacterium modesticaldum* during phototrophic and chemotrophic growth. BMC Microbiol 10: 150 10.1186/1471-2180-10-150 20497547 PMC2887804

[50] Saitou N, Nei M (1987) The neighbor-joining method: a new method for reconstructing phylogenetic trees. Mol Biol Evol 4: 406–425. 3447015 10.1093/oxfordjournals.molbev.a040454

[51] Tamura K, Stecher G, Peterson D, Filipski A, Kumar S (2013) MEGA6: Molecular Evolutionary Genetics Analysis version 6.0. Mol Biol Evol 30: 2725–2729. 10.1093/molbev/mst197 24132122 PMC3840312

[52] Mukai M, Savard PY, Ouellet H, Guertin M, Yeh SR (2002) Unique ligand-protein interactions in a new truncated hemoglobin from *Mycobacterium tuberculosis* . Biochemistry 41: 3897–3905. 11900532 10.1021/bi0156409

[53] Couture M, Burmester T, Hankeln T, Rousseau DL (2001) The heme environment of mouse neuroglobin. Evidence for the presence of two conformations of the heme pocket. J Biol Chem 276: 36377–36382. 11473111 10.1074/jbc.M103907200

[54] Saikin SK, Khin Y, Huh J, Hannout M, Wang Y, et al (2014) Chromatic acclimation and population dynamics of green sulfur bacteria grown with spectrally tailored light. Sci Rep 4: 5057 10.1038/srep05057 24862580 PMC4033924

[55] Ouellet H, Milani M, LaBarre M, Bolognesi M, Couture M, et al (2007) The roles of Tyr(CD1) and Trp(G8) in *Mycobacterium tuberculosis* truncated hemoglobin O in ligand binding and on the heme distal site architecture. Biochemistry 46: 11440–11450. 17887774 10.1021/bi7010288

[56] Feis A, Lapini A, Catacchio B, Brogioni S, Foggi P, et al (2008) Unusually strong H-bonding to the heme ligand and fast geminate recombination dynamics of the carbon monoxide complex of *Bacillus subtilis* truncated hemoglobin. Biochemistry 47: 902–910. 18154317 10.1021/bi701297f

[57] Ouellet H, Ouellet Y, Richard C, Labarre M, Wittenberg B, et al (2002) Truncated hemoglobin HbN protects Mycobacterium bovis from nitric oxide. Proc Natl Acad Sci U S A 99: 5902–5907. 11959913 10.1073/pnas.092017799 PMC122874

[58] Ouellet H, Juszczak L, Dantsker D, Samuni U, Ouellet YH, et al (2003) Reactions of *Mycobacterium tuberculosis* truncated hemoglobin O with ligands reveal a novel ligand-inclusive hydrogen bond network. Biochemistry 42: 5764–5774. 12741834 10.1021/bi0270337

[59] Kitagawa T, Ozaki Y (1987) Infrared and raman spectra of metalloporphyrins. Struct Bonding 64: 71–114.

[60] Spiro TG, Li XY (1988) Resonance Raman spectroscopy of metalloproteins. In Biological Applications of Raman Spectroscopy. (Ed.) Spiro T. G., John Wiley, New York pp. 39–96.

[61] Rousseau DL, Ondrias MR (1983) Resonance Raman scattering studies of the quaternary structure transition in hemoglobin. Annu Rev Biophys Bioeng 12: 357–380. 6347041 10.1146/annurev.bb.12.060183.002041

[62] Das TK, Couture M, Lee HC, Peisach J, Rousseau DL, et al (1999) Identification of the ligands to the ferric heme of *Chlamydomonas* chloroplast hemoglobin: evidence for ligation of tyrosine-63 (B10) to the heme. Biochemistry 38: 15360–15368. 10563822 10.1021/bi991237e

[63] Egawa T, Yeh SR (2005) Structural and functional properties of hemoglobins from unicellular organisms as revealed by resonance Raman spectroscopy. J Inorg Biochem 99: 72–96. 15598493 10.1016/j.jinorgbio.2004.10.017

[64] Couture M, Das TK, Lee HC, Peisach J, Rousseau DL, et al (1999) *Chlamydomonas* chloroplast ferrous hemoglobin. Heme pocket structure and reactions with ligands. J Biol Chem 274: 6898–6910. 10066743 10.1074/jbc.274.11.6898

[65] Goodin DB, McRee DE (1993) The Asp-His-Fe triad of cytochrome *c* peroxidase controls the reduction potential, electronic structure, and coupling of the tryptophan free radical to the heme. Biochemistry 32: 3313–3324. 8384877

[66] Mukai M, Mills CE, Poole RK, Yeh SR (2001) Flavohemoglobin, a globin with a peroxidase-like catalytic site. J Biol Chem 276: 7272–7277. 11092893 10.1074/jbc.M009280200

[67] Ermler U, Siddiqui RA, Cramm R, Friedrich B (1995) Crystal structure of the flavohemoglobin from *Alcaligenes eutrophus* at 1.75 A resolution. EMBO J 14: 6067–6077. 8557026 10.1002/j.1460-2075.1995.tb00297.x PMC394731

[68] Lu C, Mukai M, Lin Y, Wu G, Poole RK, et al (2007) Structural and functional properties of a single domain hemoglobin from the food-borne pathogen *Campylobactor jejuni* . J Biol Chem 282: 25917–25928. 17606611 10.1074/jbc.M704415200

[69] Dawson JH (1988) Probing structure-function relations in heme-containing oxygenases and peroxidases. Science 240: 433–439. 3358128 10.1126/science.3358128

[70] Samuni U, Ouellet Y, Guertin M, Friedman JM, Yeh SR (2004) The absence of proximal strain in the truncated hemoglobins from *Mycobacterium tuberculosis* . J Am Chem Soc 126: 2682–2683. 14995168 10.1021/ja038093i

[71] Chen Z, Ost TW, Schelvis JP (2004) Phe393 mutants of cytochrome P450 BM3 with modified heme redox potentials have altered heme vinyl and propionate conformations. Biochemistry 43: 1798–1808. 14967021 10.1021/bi034920g

[72] Feehily C, Karatzas KA (2013) Role of glutamate metabolism in bacterial responses towards acid and other stresses. J Appl Microbiol 114: 11–24. 10.1111/j.1365-2672.2012.05434.x 22924898

[73] Coyle JT, Puttfarcken P (1993) Oxidative stress, glutamate, and neurodegenerative disorders. Science 262: 689–695. 7901908 10.1126/science.7901908

[74] Ramond E, Gesbert G, Rigard M, Dairou J, Dupuis M, et al (2014) Glutamate utilization couples oxidative stress defense and the tricarboxylic acid cycle in *Francisella* phagosomal escape. PLoS Pathog 10: e1003893 10.1371/journal.ppat.1003893 24453979 PMC3894225

[75] Haghighi AZ, Maples KR (1996) On the mechanism of the inhibition of glutamine synthetase and creatine phosphokinase by methionine sulfoxide. J Neurosci Res 43: 107–111. 8838581 10.1002/jnr.490430114

[76] Yeh SR (2004) A novel intersubunit communication mechanism in a truncated hemoglobin from *Mycobacterium tuberculosis* . J Phys Chem B 108: 1478–1484.

[77] Lu C, Egawa T, Wainwright LM, Poole RK, Yeh SR (2007) Structural and functional properties of a truncated hemoglobin from a food-borne pathogen *Campylobacter jejuni* . J Biol Chem 282: 13627–13636. 17339325 10.1074/jbc.M609397200

[78] Ouellet H, Ranguelova K, Labarre M, Wittenberg JB, Wittenberg BA, et al (2007) Reaction of *Mycobacterium tuberculosis* truncated hemoglobin O with hydrogen peroxide: evidence for peroxidatic activity and formation of protein-based radicals. J Biol Chem 282: 7491–7503. 17218317 10.1074/jbc.M609155200

[79] Torge R, Comandini A, Catacchio B, Bonamore A, Botta B, et al (2009) Peroxidase-like activity of *Thermobifida fusca* hemoglobin: The oxidation of dibenzylbutanolide. J Mol Catal B: Enzym 61: 303–308.

[80] Everse J, Johnson MC, Marini MA (1994) Peroxidative activities of hemoglobin and hemoglobin derivatives. Methods Enzymol 231: 547–561. 8041276 10.1016/0076-6879(94)31038-6

[81] Lafay B, Ruimy R, de Traubenberg CR, Breittmayer V, Gauthier MJ, et al (1995) *Roseobacter algicola* sp. nov., a new marine bacterium isolated from the phycosphere of the toxin-producing dinoflagellate *Prorocentrum lima* . Int J Syst Bacteriol 45: 290–296. 7537061 10.1099/00207713-45-2-290

[82] Ruiz-Ponte C, Cilia V, Lambert C, Nicolas JL (1998) *Roseobacter gallaeciensis* sp. nov., a new marine bacterium isolated from rearings and collectors of the scallop *Pecten maximus* . Int J Syst Bacteriol 48 Pt 2: 537–542. 10.1099/00207713-48-2-537 9731295

[83] Dai X, Wang BJ, Yang QX, Jiao NZ, Liu SJ (2006) *Yangia pacifica* gen. nov., sp. nov., a novel member of the *Roseobacter* clade from coastal sediment of the East China Sea. Int J Syst Evol Microbiol 56: 529–533. 16514022 10.1099/ijs.0.64013-0

[84] Thiel V, Brinkhoff T, Dickschat JS, Wickel S, Grunenberg J, et al (2010) Identification and biosynthesis of tropone derivatives and sulfur volatiles produced by bacteria of the marine *Roseobacter* clade. Org Biomol Chem 8: 234–246. 10.1039/b909133e 20024154

[85] Brinkhoff T, Bach G, Heidorn T, Liang L, Schlingloff A, et al (2004) Antibiotic production by a *Roseobacter* clade-affiliated species from the German Wadden Sea and its antagonistic effects on indigenous isolates. Appl Environ Microbiol 70: 2560–2565. 15066861 10.1128/AEM.70.4.2560-2565.2003 PMC383154

[86] Faulkner DJ (2000) Marine natural products. Nat Prod Rep 17: 7–55. 10714898 10.1039/a809395d

[87] Wagner-Dobler I, Rheims H, Felske A, El-Ghezal A, Flade-Schroder D, et al (2004) *Oceanibulbus indolifex* gen. nov., sp. nov., a North Sea alphaproteobacterium that produces bioactive metabolites. Int J Syst Evol Microbiol 54: 1177–1184. 15280288 10.1099/ijs.0.02850-0

[88] Bruhn JB, Gram L, Belas R (2007) Production of antibacterial compounds and biofilm formation by *Roseobacter* species are influenced by culture conditions. Appl Environ Microbiol 73: 442–450. 17098910 10.1128/AEM.02238-06 PMC1796973

[89] Milani M, Pesce A, Ouellet H, Guertin M, Bolognesi M (2003) Truncated hemoglobins and nitric oxide action. IUBMB Life 55: 623–627. 14711009 10.1080/15216540310001628708

[90] Gardner PR (2005) Nitric oxide dioxygenase function and mechanism of flavohemoglobin, hemoglobin, myoglobin and their associated reductases. J Inorg Biochem 99: 247–266. 15598505 10.1016/j.jinorgbio.2004.10.003

[91] Elvers KT, Turner SM, Wainwright LM, Marsden G, Hinds J, et al (2005) NssR, a member of the Crp-Fnr superfamily from *Campylobacter jejuni*, regulates a nitrosative stress-responsive regulon that includes both a single-domain and a truncated haemoglobin. Mol Microbiol 57: 735–750. 16045618 10.1111/j.1365-2958.2005.04723.x

[92] Pathania R, Navani NK, Gardner AM, Gardner PR, Dikshit KL (2002) Nitric oxide scavenging and detoxification by the *Mycobacterium tuberculosis* haemoglobin, HbN in *Escherichia coli* . Mol Microbiol 45: 1303–1314. 12207698 10.1046/j.1365-2958.2002.03095.x

[93] Ascenzi P, De Marinis E, Coletta M, Visca P (2008) H2O2 and (.)NO scavenging by *Mycobacterium leprae* truncated hemoglobin O. Biochem Biophys Res Commun 373: 197–201. 10.1016/j.bbrc.2008.05.168 18544337

[94] Zumft WG (1997) Cell biology and molecular basis of denitrification. Microbiol Mol Biol Rev 61: 533–616. 9409151 10.1128/mmbr.61.4.533-616.1997 PMC232623

[95] Tang KH, Tang YJ, Blankenship RE (2011) Carbon metabolic pathways in phototrophic bacteria and their broader evolutionary implications. Front Microbiol 2: 165 10.3389/fmicb.2011.00165 21866228 PMC3149686

[96] Yurkov VV, Beatty JT (1998) Aerobic anoxygenic phototrophic bacteria. Microbiol Mol Biol Rev 62: 695–724. 9729607 10.1128/mmbr.62.3.695-724.1998 PMC98932

[97] Schweizer M, Richter C (1994) Nitric oxide potently and reversibly deenergizes mitochondria at low oxygen tension. Biochem Biophys Res Commun 204: 169–175. 7945356 10.1006/bbrc.1994.2441

[98] Gardner AM, Helmick RA, Gardner PR (2002) Flavorubredoxin, an inducible catalyst for nitric oxide reduction and detoxification in *Escherichia coli* . J Biol Chem 277: 8172–8177. 11751865 10.1074/jbc.M110471200

[99] Tomasch J, Gohl R, Bunk B, Diez MS, Wagner-Dobler I (2011) Transcriptional response of the photoheterotrophic marine bacterium *Dinoroseobacter shibae* to changing light regimes. ISME J 5: 1957–1968. 10.1038/ismej.2011.68 21654848 PMC3223308

[100] Brennan AM, Suh SW, Won SJ, Narasimhan P, Kauppinen TM, et al (2009) NADPH oxidase is the primary source of superoxide induced by NMDA receptor activation. Nat Neurosci 12: 857–863. 10.1038/nn.2334 19503084 PMC2746760

[101] Johnson EA, Rice SL, Preimesberger MR, Nye DB, Gilevicius L, et al (2014) Characterization of THB1, a *Chlamydomonas reinhardtii* truncated Hemoglobin: Linkage to nitrogen metabolism and identification of lysine as the distal heme ligand. Biochemistry 53: 4573–4589. 10.1021/bi5005206 24964018 PMC4108185

