# Supplementary figures and images for: Peroxidase Activity and Involvement in the Oxidative Stress Response of Roseobacter denitrificans Truncated Hemoglobin
Source: PLoS One. 2015 Feb 6;10(2):e0117768. doi: 10.1371/journal.pone.0117768 (PMC4319818; doi:10.1371/journal.pone.0117768)

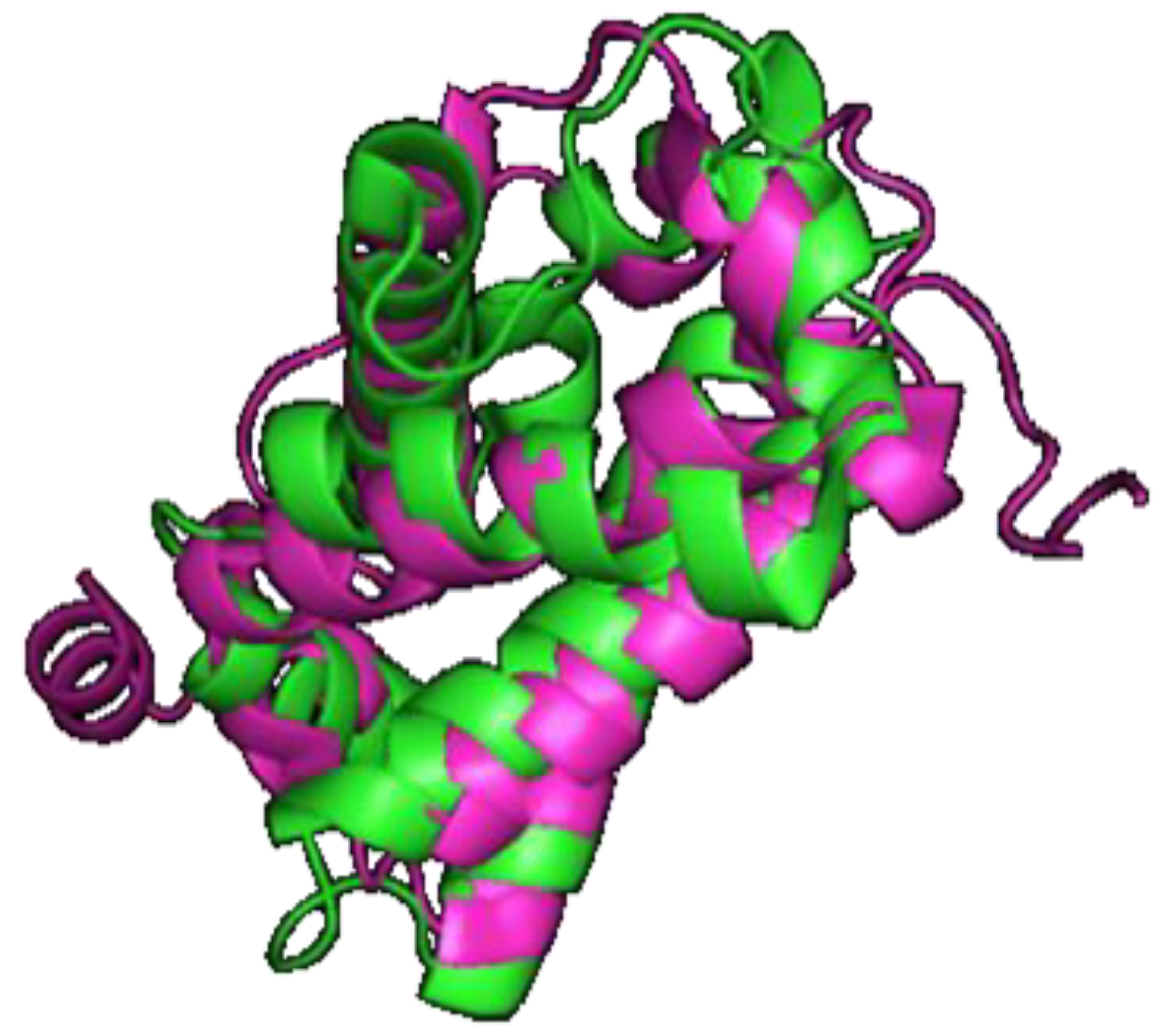

Supplement: S1 Fig — (TIF) [file pone.0117768.s001.tif]

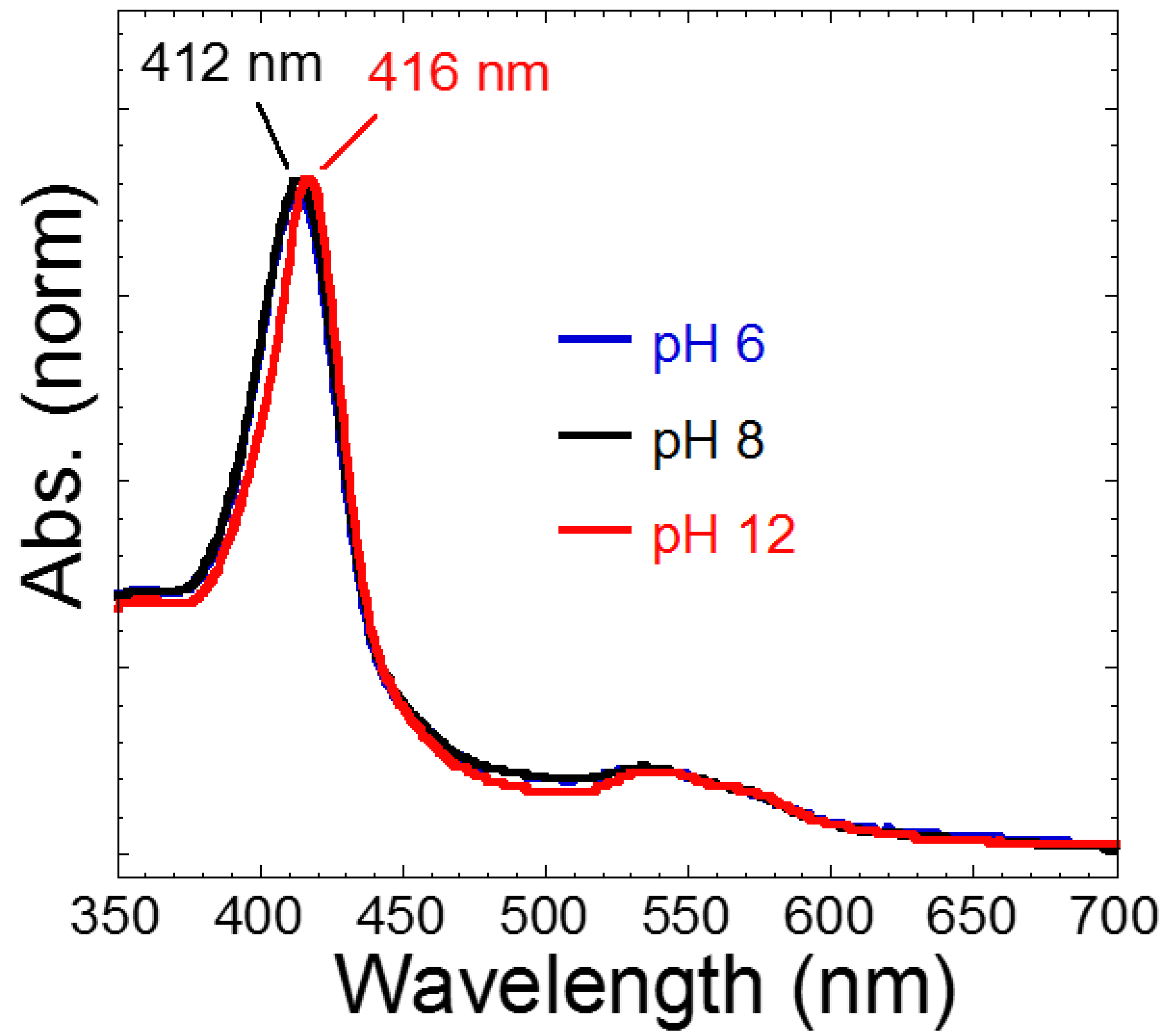

Supplement: S2 Fig — (TIF) [file pone.0117768.s002.tif]
